# Toward a CRISPR-based mouse model of *Vhl*-deficient clear cell kidney cancer: Initial experience and lessons learned

**DOI:** 10.1073/pnas.2408549121

**Published:** 2024-10-04

**Authors:** Laura A. Stransky, Wenhua Gao, Laura S. Schmidt, Kevin Bi, Christopher J. Ricketts, Vijyendra Ramesh, Amy James, Simone Difilippantonio, Lilia Ileva, Joseph D. Kalen, Baktiar Karim, Albert Jeon, Tamara Morgan, Andrew C. Warner, Sevilay Turan, Joanne Unite, Bao Tran, Sulbha Choudhari, Yongmei Zhao, Douglas E. Linn, Changhong Yun, Sripriya Dhandapani, Vaishali Parab, Elaine M. Pinheiro, Nicole Morris, Lixia He, Sean M. Vigeant, Jean-Christophe Pignon, Maura Sticco-Ivins, Sabina Signoretti, Eliezer M. Van Allen, W. Marston Linehan, William G. Kaelin

**Affiliations:** ^a^Division of Molecular and Cellular Oncology, Department of Medical Oncology, Dana-Farber Cancer Institute, Harvard Medical School, Boston, MA 02215; ^b^Urologic Oncology Branch, Center for Cancer Research, National Cancer Institute, NIH, Bethesda, MD 20892; ^c^Basic Science Program, Frederick National Laboratory for Cancer Research, Frederick, MD 21702; ^d^Division of Population Sciences, Department of Medical Oncology, Dana-Farber Cancer Institute, Boston, MA 02115; ^e^Center for Cancer Genomics, Dana-Farber Cancer Institute, Boston, MA 02115; ^f^Broad Institute of Harvard and Massachusetts Institute of Technology, Cambridge, MA 02142; ^g^Animal Research Technical Support, Frederick National Laboratory for Cancer Research, Frederick, MD 21702; ^h^Small Animal Imaging Program, Frederick National Laboratory for Cancer Research, Frederick, MD 21702; ^i^Molecular Histopathology Laboratory, Frederick National Laboratory for Cancer Research, Frederick, MD 21702; ^j^National Cancer Institute Center for Cancer Research, Sequencing Facility, Frederick National Laboratory for Cancer Research, Frederick, MD 21701; ^k^Advanced Biomedical and Computational Science, Frederick National Laboratory for Cancer Research, Frederick, MD 21701; ^l^Quantitative Biosciences, Merck & Co., Inc., Boston, MA 02115; ^m^Pharmacokinetics, Merck & Co., Inc., Boston, MA 02115; ^n^Pharmacokinetics, Merck & Co., Inc., South San Francisco, CA 94080; ^o^Discovery Oncology Merck & Co., Inc., Boston, MA 02115; ^p^Laboratory of Animal Sciences Program, Frederick National Laboratory for Cancer Research, Frederick, MD 21702; ^q^Harvard Medical School, Boston, MA 02115; ^r^Department of Pathology, Brigham and Women's Hospital, Boston, MA 02115; ^s^Department of Oncologic Pathology, Dana-Farber Cancer Institute, Boston, MA 02115; ^t^HHMI, Chevy Chase, MD 20815

**Keywords:** VHL, HIF2, ccRCC, PT2399, mouse models of ccRCC

## Abstract

CRISPR is revolutionizing the ability to do somatic gene editing in mice for the purpose of creating new cancer models. Inactivation of the *VHL* tumor suppressor gene is the signature initiating event in the most common form of kidney cancer, clear cell renal cell carcinoma (ccRCC). Such tumors are usually driven by the excessive HIF2 activity that arises when the *VHL* gene product, pVHL, is defective. Given the pressing need for a robust immunocompetent mouse model of human ccRCC, we directly injected adenovirus-associated viruses (AAVs) encoding sgRNAs against *VHL* and other known/suspected ccRCC tumor suppressor genes into the kidneys of C57BL/6 mice under conditions where Cas9 was under the control of one of two different kidney-specific promoters (*Cdh16* or *Pax*8) to induce kidney tumors. An AAV targeting *Vhl, Pbrm1, Keap1*, and *Tsc1* reproducibly caused macroscopic ccRCCs that partially resembled human ccRCC tumors with respect to transcriptome and cell of origin and responded to a ccRCC standard-of-care agent, axitinib. Unfortunately, these tumors, like those produced by earlier genetically engineered mouse ccRCCs, are HIF2 independent.

## Significance

Clear cell renal cell carcinoma (ccRCC) is the most common form of kidney cancer, which is one of the ten most common cancers in the developed world. Most ccRCCs are caused by inactivation of the *VHL* tumor suppressor gene and the resulting accumulation of the HIF2 transcription factor. However, there are currently no immunocompetent mouse models of *VHL^−/−^*, HIF2 dependent, ccRCC. Although we succeeded in generating *VHL−/−*ccRCCs in mice using somatic gene editing of *VHL* and other kidney cancer suppressor genes that strongly resemble human ccRCC tumors in some respects, these lesions were not HIF2 dependent. We describe herein potential explanations and paths forward.

In 2023, there were an estimated 81,800 new cases of renal cell carcinoma (RCC) and an estimated 14,890 deaths in the United States due to this disease, with twice as many cases in men as in women (1). Clear cell RCC (ccRCC) is the most common histologic subtype of RCC, accounting for ~75% of cases. Inactivation of the *von-Hippel Lindau* (*VHL)* tumor suppressor gene is the initiating or “truncal” event in nearly 90% of sporadic clear cell renal cell carcinomas and in all ccRCCs that develop in the setting of the hereditary cancer syndrome, von Hippel–Lindau (VHL) disease (2–6). pVHL is the substrate recognition component of an E3 ubiquitin ligase complex that targets the alpha subunits of the hypoxia-inducible factor (HIF) transcription factors for proteasomal degradation when oxygen is available (7). Deregulation of the HIF pathway, and particularly HIF2, causes the cellular proliferations observed after *VHL* inactivation in mice (8–10) and drives pVHL-defective tumor formation in mouse xenograft models of *VHL^−/−^* ccRCC (11–18).

Although *VHL* inactivation is a critical first step in ccRCC pathogenesis, it is not sufficient to cause ccRCC. ccRCCs harbor additional cooperating mutations, including intragenic mutations and nonfocal gains/losses of specific chromosomal regions (19, 20). Stereotypical among the copy number changes are loss of chromosome 3p, 9p, and 14q and gain of chromosome 5q. Human chromosome 3p harbors the *VHL* gene as well as the ccRCC suppressors *PBRM1*, *SETD2*, and *BAP1*, all three of which encode epigenetic regulators. *CDKN2A* is a likely target of chromosome 9p deletions in ccRCC (19). A number of putative ccRCC suppressors exist on chromosome 14q, including *HIF1A* (21) and *ARG*2 (22), and several putative ccRCC oncoproteins have been identified on chromosome 5q, including *SQSTM1* (23, 24). *SQSTM1* encodes p62, which is believed to activate multiple downstream effectors, including mTOR and NRF2 (25). Notably, a subset of ccRCCs have intragenic mutations that inactivate upstream inhibitors of mTOR, such as inactivation of *PTEN* or *TSC1* (or *TSC2*) (20). In addition, a small subset of ccRCCs harbor amplification of *NFEL2*, which encodes NRF2, or mutational inactivation of the NRF2 inhibitor *KEAP1*. Inactivation of KEAP1 also occurs in many ccRCCs due to promoter hypermethylation (26).

Although surgery is often curative when ccRCC is detected early at a localized stage, many patients relapse after surgery or have advanced-stage disease when diagnosed. Consequently, there is a need to develop effective systemic therapies. Agents that target the HIF-responsive gene product VEGF and its receptor, and agents that inhibit the mTOR pathway, are approved for ccRCC treatment (27). Not all ccRCC patients respond to these agents, however, and those that do will almost invariably progress. Immune checkpoint inhibitors that activate the adaptive immune system to target and kill cancer cells, such as antibodies against CTLA4, PD-1, or PD-L1, have been elevated to front-line therapy for treatment of metastatic RCC (28). Only a subset of ccRCC patients respond to immune checkpoint inhibitors, however, and they can produce significant autoimmune toxicities. A first-in-class HIF2 inhibitor, Belzutifan, was recently approved for the treatment of VHL disease-associated neoplasms, including ccRCC, and for the treatment of sporadic ccRCC (29, 30) (https://www. fda.gov/drugs/resources-information- approved- drugs/fda-approves-belzutifan-advanced-renal-cell-carcinoma). Virtually all of the ccRCCs in the setting of VHL disease arrested or shrank when treated with Belzutifan in the trial that led to its approval, suggesting that all *VHL*^−/−^ ccRCCs are initially HIF2-dependent (29). In contrast, many sporadic, advanced *VHL*^−/−^ ccRCCs treated with Belzutifan after the failure of standard-of-care agents are seemingly HIF2-independent, as are a subset of *VHL*^−/−^ ccRCC cell lines (15, 16, 31) (https://depmap.org/). This suggests that *VHL*^−/−^ ccRCCs can evolve toward HIF2-independence over time, perhaps aided by selection pressures imparted by kidney cancer therapies.

Preclinical models of ccRCC are critical research tools for testing potential therapeutic agents, especially models that recapitulate the tumor microenvironment in human RCC. Patient-derived and RCC cell line-derived xenograft models are the most frequently utilized preclinical systems in RCC research, but these models necessarily employ immunocompromised mice to avoid tumor rejection, thereby prohibiting their use in evaluating immune checkpoint inhibitors. A number of genetically engineered mouse models (GEMM) for *VHL^−/−^* ccRCC have been created in which the *VHL* gene, together with additional tumor suppressor genes, have been inactivated in the germline (32–42). An advantage of these models is that they are immunocompetent. On the other hand, these models have suffered from a number of shortcomings. Some are characterized by the occurrence of bilateral and multifocal tumors and cysts that compromise kidney function at an early age, which limits their utility as models for drug treatment. Others produce only small tumors with long latencies and low penetrance. Some of the published mouse models of *VHL^−/−^* cRCC have incorporated mutations that are rarely observed in human ccRCCs. Finally, the tumors produced in these models have invariably been HIF2 independent when tested.

CRISPR/Cas9-based gene editing has been used in mice to generate tumors by multiple simultaneous somatic gene editing of selected genes in the lung (43, 44), liver (45), brain (46), and kidney (42, 47). These models often utilized a mouse strain that conditionally expresses Cas9 upon Cre-mediated excision of a “Lox-Stop-Lox” cassette and virally delivered sgRNAs against the target genes of interest. We used this approach to create mouse models of *VHL^−/−^* ccRCC.

## Results and Discussion

We designed CRISPR sgRNAs against murine *Vhl, Pbrm1, Tsc1, Setd2, Keap1, Cdkn2a, Bap1*, and *Hif1a.* We individually expressed each sgRNA to be tested in murine 3T3 fibroblasts using a lentiviral vector that also expresses Cas9 and monitored gene targeting efficiency by immunoblotting for the protein product of the sgRNA target and/or a pharmacodynamic marker for that protein product.

**Fig. 1.**
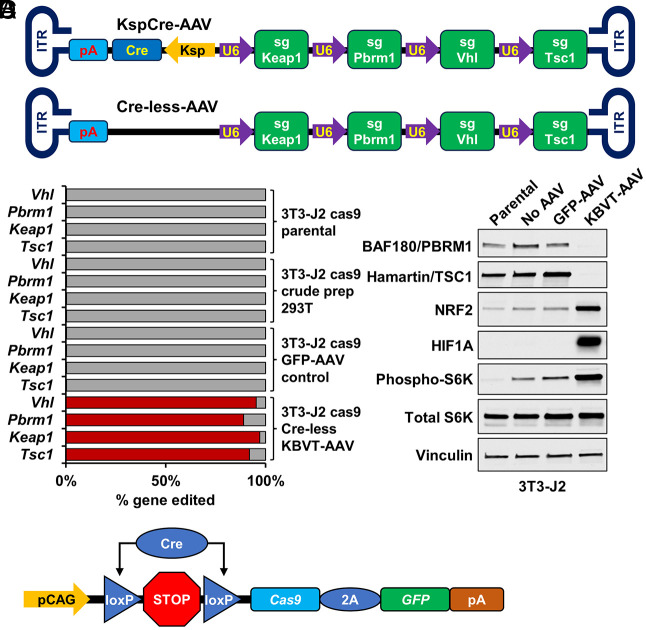
CRISPR/Cas9 gene editing strategy for generating a syngeneic *Vhl*-deficient ccRCC mouse model. (*A*) Schematic representation of Ksp-Cre AAV and Cre-less AAV vectors. (*B*) % gene editing of *Vhl, Pbrm1, Keap1*, and *Tsc1* in 3T3-J2 Cas9 parental cells, 3T3-J2 Cas9 crude prep, 3T3-J2 Cas9 cells with GFP-AAV (control), 3T3-J2 Cas9 cells with AAV carrying *Vhl, Pbrm1, Keap1*, and *Tsc1* sgRNAs. (*C*) Immunoblot of 3T3-J2 Cas9 cell preps shown in (*B*) indicating effective gene editing. (*D*) Lox-STOP-Lox-Cas9-GFP cassette in genetically modified Cas9/+ mice denoting Cre recombinase excision of loxP flanked STOP sequence.

Next, we created adenovirus-associated virus (AAV) vectors that incorporated a *Vhl* sgRNA and up to 3 additional sgRNAs against the known or suspected ccRCC suppressor genes listed above. We used two different AAV vectors, one of which expressed Cre recombinase under the kidney-specific *Cdh16*(KSP) promoter (“Ksp-Cre AAV”) and one of which did not (“Cre-less AAV”) (Fig. 1*A*). These AAVs were then used to infect murine 3T3-J2 cells that stably expressed Cas9. Gene editing was confirmed by sequencing the target genes and by immunoblotting as above (see, e.g., Fig. 1 *B* and *C*).

For each sgRNA combination to be tested in vivo, we orthotopically injected the corresponding AAV into the kidneys of immunocompetent C57BL/6 mice. The Ksp-Cre AAVs were injected into a previously reported mouse strain [Gt(ROSA) 26Sor^tm1(CAG-cas9*,-EGFP)Fezh^; common name ROSA26-LSL-Cas9- EGFP knockin, hereafter LSL-Cas9/+ for simplicity] that expresses both Cas9 and GFP after Cre-mediated excision of a Lox-Stop-Lox cassette (43) (Fig. 1*D*). The Cre-less AAVs were injected into LSL-Cas9/+ mice that also transgenically expressed Cre recombinase under the control of the *Pax8* promoter (Pax8 Cre/+) allowing conditional expression of Cas9 in *Pax8* expressing cells (48). Pax8 is expressed in human renal epithelial cells in all segments of renal tubules from the proximal tubules to the renal papillae and in the parietal cells of Bowman’s capsule (49), and in proximal tubules, collecting ducts, inner and outer medulla, and renal papilla in the adult mouse kidney (50). Moreover, PAX8 cooperates with HIF2 to promote ccRCC (51).

We noticed that renal tumors only developed, as detected by serial MRIs, using AAVs that included a *Keap1* sgRNA (*SI Appendix*, Table S1) and that the most reliable combination of sgRNAs for producing renal tumors was *Keap1, Pbrm1, Vhl, and Tsc1.* We therefore chose to study this combination further. For simplicity, the “Ksp-Cre AAV model” and “Cre-less AAV model” will be used below when describing tumors caused by the Ksp-Cre AAV and Cre-less AAV, respectively, expressing sgRNAs against *Keap1*, *Pbrm1*, *Vhl*, and *Tsc1*.

**Fig. 2.**
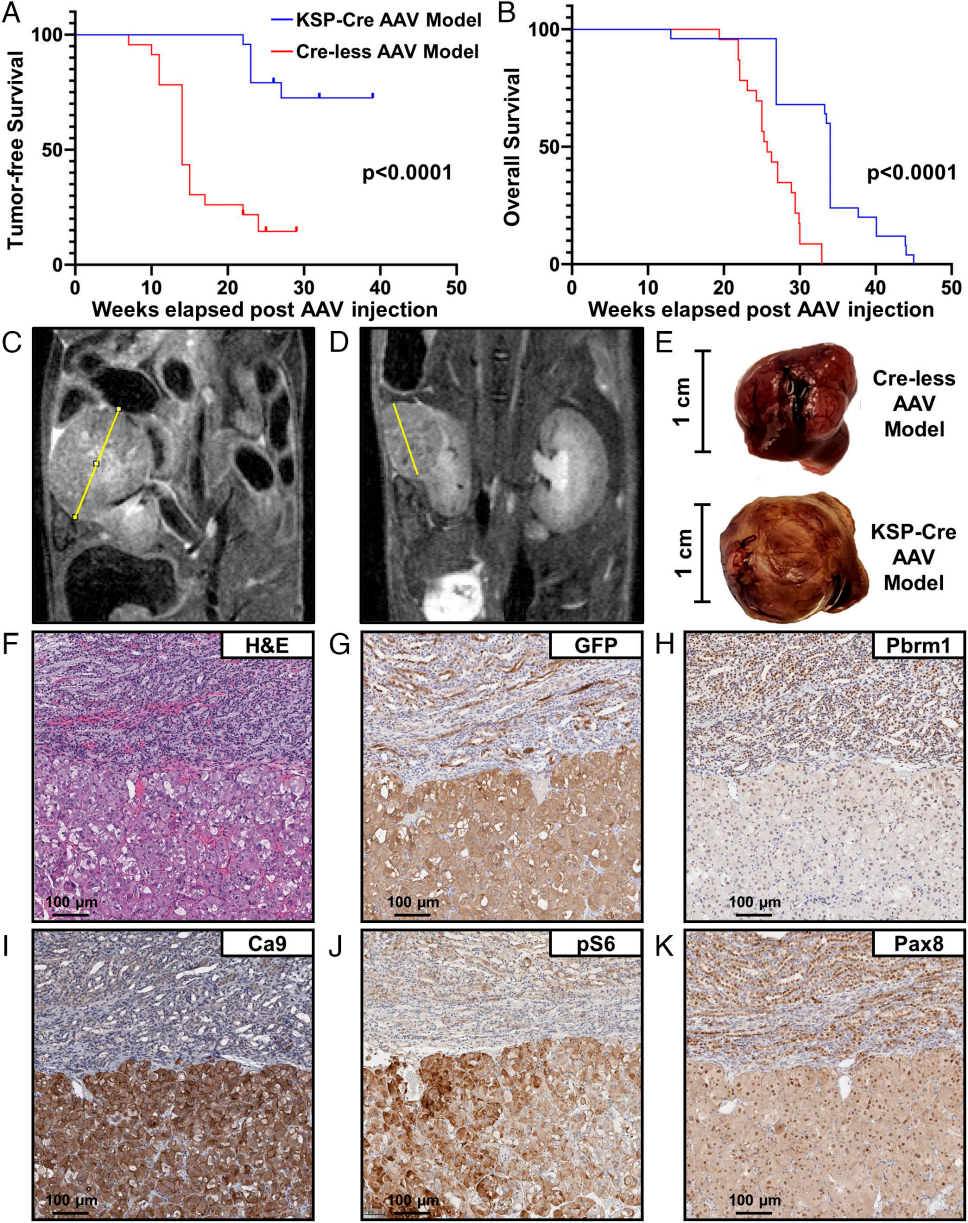
Combined loss of *Vhl, Pbrm1, Keap1*, and *Tsc1* is sufficient to drive ccRCC development. (*A*) Tumor-free survival of Ksp-Cre model vs. Cre-less AAV model. (*B*) Comparative overall survival of Ksp-Cre AAV model vs. Cre-less AAV model. N = 24 mice for KSP-Cre AAV model; n = 23 mice for Cre-less AAV model. Mice that died for reasons unrelated to tumor burden were censored. *P* value determined by the log-rank test, *P* < 0.0001. Tumor visualization by MRI in (*C*) Ksp-Cre AAV model and (*D*) Cre-less AAV model. Representative images of renal tumors in (*E*) Ksp-Cre AAV-injected LSL-Cas9/+ mice and Cre-less AAV-injected LSL-Cas9/+, Pax8 Cre mice. Immunohistochemistry of markers indicating targeted gene editing. (*F*) H&E staining of Cre-less AAV model tumor with adjacent normal kidney (upper region) showing a mixed clear cell and eosinophilic histology. Immunohistochemical staining of Cre-less AAV model for (*G*) GFP, (*H*) Baf180 (Pbrm1), (*I*) Ca9, (*J*) phospho-S6 (mTOR readout), and (*K*) Pax8. (Scale bar, 100 µm.)

The Cre-less AAV model produced tumors with greater penetrance (83%) and shorter latency (9 wk) compared to the Ksp-Cre AAV model (33% penetrance and 14-wk latency) (Table 1 and Fig. 2*A*), which was reflected in the reduced overall survival of Cre-less AAV model mice when compared to the Ksp-Cre AAV model mice (50% survival, 25.7 wk vs. 34 wk, *P* < 0.0001) (Fig. 2*B*). Representative MRIs and gross images are shown in Fig. 2 *C–E*, respectively.

**Table 1.** Tumor incidence, latency, and growth rate in AAV mouse models

AAV model	KSP-Cre AAV-> LSL-Cas9/+	Creless AAV-> LSL-Cas9/+; Pax8 Cre
**No. of mice with tumor (% penetrance)**	8/24 (33%)	19/23 (83%)
**Time to initial tumor detection by MRI (range)**	14 wk (14 to 32 wk)	9 wk (9 to 20 wk)
**Mean tumor growth rate**	0.78 mm/week	0.96 mm/week

On histological examination, the tumors consisted mostly of eosinophilic cells with scattered interspersed clear cells (Ksp-Cre AAV—*SI Appendix*, Fig. S1*A*; Cre-less AAV—Fig. 2*F* and *SI Appendix*, Fig. S2 *A*–*H*). The percentage of clear cells varied between tumors as well as regionally within tumors. Clear cells were somewhat more prevalent in the Cre-less model than in the KSP-Cre model. None of the tumors fully resembled the morphological appearance of classical clear cell renal cell carcinoma. However, it is well recognized that ccRCC shows a wide spectrum of morphological features and that some cases consist predominantly of cells with eosinophilic cytoplasm (52).

In both models immunohistochemistry confirmed expression of GFP, consistent with Cre-mediated excision of the Lox-Stop-Lox cassette in the LSL-Cas9/+ mice, loss of staining for the *Pbrm1* gene product BAF180, and likely inactivation of *Vhl* and *Tsc1* as evidenced by increased staining for the HIF target Ca9 and the mTOR target phospho-S6, respectively (Fig. 2 *G*–*J* and *SI Appendix*, Fig. S1 *B*–*E*). We confirmed that Pax8, which is used to drive Cre recombinase expression in the Cre-less model, was expressed throughout the kidney as expected (Fig. 2*K*). The staining for phospho-S6 was spatially heterogeneous within tumors in both models. The explanation and significance of this finding are not clear.

Due to the greater penetrance and shorter latency for tumor development, we used the Cre-less model for tumor characterization and efficacy studies. We used deep sequencing of tumor DNA to more precisely measure the extent of CRISPR/Cas9 gene editing of *Keap1, Pbrm1, Vhl*, and *Tsc1* in this model. We consistently achieved on average greater than 56% gene editing of *Vhl*, 65% gene editing for *Keap1*, 61% gene editing for *Tsc1*, but more reduced gene editing (on average, 17%) for *Pbrm1* (*SI Appendix*, Fig. S3 and Table S2). Copy number variation was queried by whole genome sequencing, but no significant chromosomal losses/gains were detected.

**Fig. 3.**
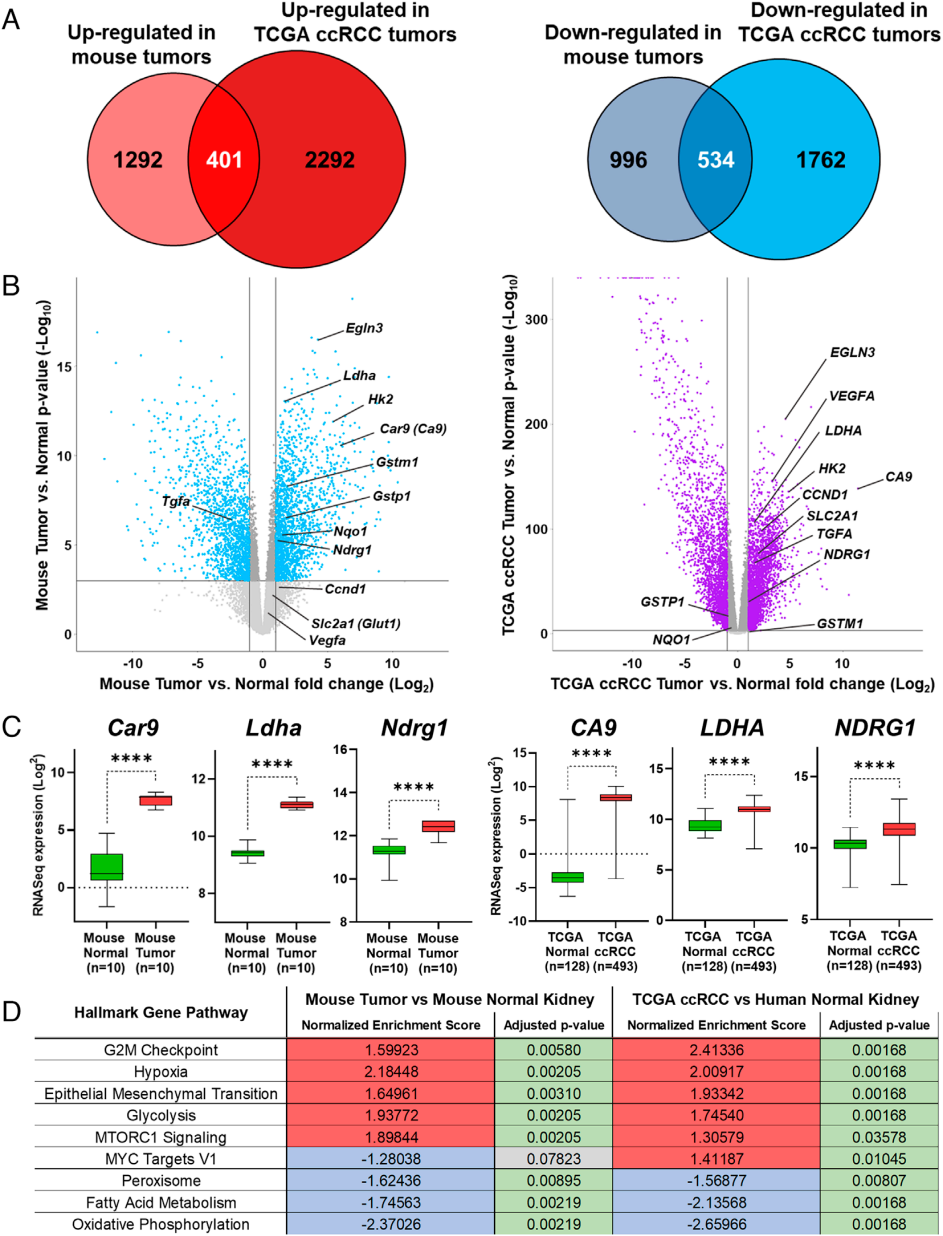
Comparative gene expression analysis of Cre-less AAV model kidney tumors and TCGA human ccRCC. (*A*) Venn diagrams showing up-regulated and down-regulated genes in Cre-less AAV model tumors vs. TCGA ccRCC as shown in volcano plots. (*B*) Volcano plots displaying up-regulated and down-regulated genes in Cre-less AAV kidney tumors vs. normal mouse kidney (*Left* plot) and in TCGA ccRCC vs. normal human kidney (*Right* plot). (*C*) Hypoxia-related gene expression in Cre-less AAV mouse tumor vs. normal mouse kidney (*Left* panels) and in TCGA ccRCC vs. normal human kidney (*Right* panels). (*D*) GSEA showing significantly up-or down-regulated pathways in Cre-less AAV model kidney tumor vs normal mouse kidney and TCGA ccRCC vs normal human kidney.

To determine whether the tumors faithfully represented molecular hallmarks of human ccRCC tumors, 10 Cre-less AAV mouse tumors and 10 normal mouse kidneys from an initial cohort of Cre-less model mice were subjected to RNA sequencing analysis and the gene expression profile was compared to the Cancer Genome Atlas (TCGA) KIRC dataset (53). The mouse tumors demonstrated a distinct transcriptome profile in comparison to normal mouse kidney with a total of 1,693 genes being up-regulated in mouse tumors (Log_2_ FC > 1, *P* < 0.001) in comparison to 2,693 up-regulated genes in the TCGA ccRCC cohort compared to normal human kidney (Log_2_ FC > 1, *P* < 0.001), with 401 genes shared between these tumors across species (Fig. 3*A* and *SI Appendix*, Fig. S4). Similarly, a total of 1,530 genes were down-regulated in the mouse tumors relative to normal mouse kidney (Log_2_ FC < -1, *P* < 0.001) compared to 2,296 down-regulated genes in the TCGA dataset (Log_2_ FC < -1, *P* < 0.001), with 534 down-regulated genes shared between the different species (Fig. 3*A*). The mouse tumors demonstrated reduced expression of *Vhl* and *Tsc1*, but little change in the expression of *Pbrm1* (*SI Appendix*, Fig. S5). The latter is consistent with the low levels of *Pbrm1* editing. *Keap1* expression levels were increased in tumors despite efficient gene editing, possibly due to positive feedback resulting from loss of gene activity (*SI Appendix*, Fig. S5). Both mouse and human renal tumors displayed elevated expression of known HIF target genes (*Egln3/EGLN3, Ldha/LDHA*, *Hk2/HK2, Car9/CA9, Ndrg1/NDRG1*; Fig. 3 *B* and *C*). Although *Keap1* gene expression was not reduced in the mouse tumors compared to normal mouse kidney (*SI Appendix*, Fig. S5), increased expression of Nrf2 transcriptional target genes *Nqo1, Gstm1*, and *Gstp1* was observed in the mouse tumors, consistent with loss of Keap1 activity (*SI Appendix*, Fig. S6). Notably, however, we did not see evidence of NRF2 activation in the human ccRCC TCGA dataset (Fig. 3*B* and *SI Appendix*, Fig. S6). This latter observation calls into question the importance of NRF2 with respect to human ccRCC pathogenesis.

Gene set enrichment analysis (GSEA) of up- and down-regulated genes in the mouse and human renal tumors compared to their respective normal kidney parenchyma revealed high enrichment scores for pathways involved in cell cycle G2M checkpoint, hypoxia, glycolysis, and mTOR signaling (Fig. 3*D*). The enrichment for hypoxia-induced mRNAs is consistent with genetic ablation of *VHL*, but could also reflect the bona fide hypoxia that invariably occurs as solid tumors expand. Both mouse and human renal tumors displayed negative enrichment scores for genes associated with peroxisome biogenesis, fatty acid metabolism, and oxidative phosphorylation. Interestingly, a negative enrichment score was obtained for the gene pathway related to *Myc* targets in mouse tumors, but received a positive enrichment score in the TCGA ccRCC cohort (Fig. 3*D*). In this regard, HIF1 has been shown to inhibit Myc while HIF2 cooperates with Myc (54). We have not, with the available reagents, been able to detect Hif2α by immunoblot or immunohistochemical analysis in our mouse tumors. Moreover, the mRNA levels for *Epas1*, which encodes Hif2α, were lower in our mouse renal tumors than in normal kidney as determined by qRT-PCR (*SI Appendix*, Fig. S7*A*). Since *Epas1* is only expressed in a minority of normal renal cells, we conclude that the tumors produced by the Ksp-Cre AAV and Cre-less AAV models are HIF2 negative, in contrast to human VHL-defective ccRCCs.

### Determining the Cell of Origin of Cre-less AAV Model Tumors.

**Fig. 4.**
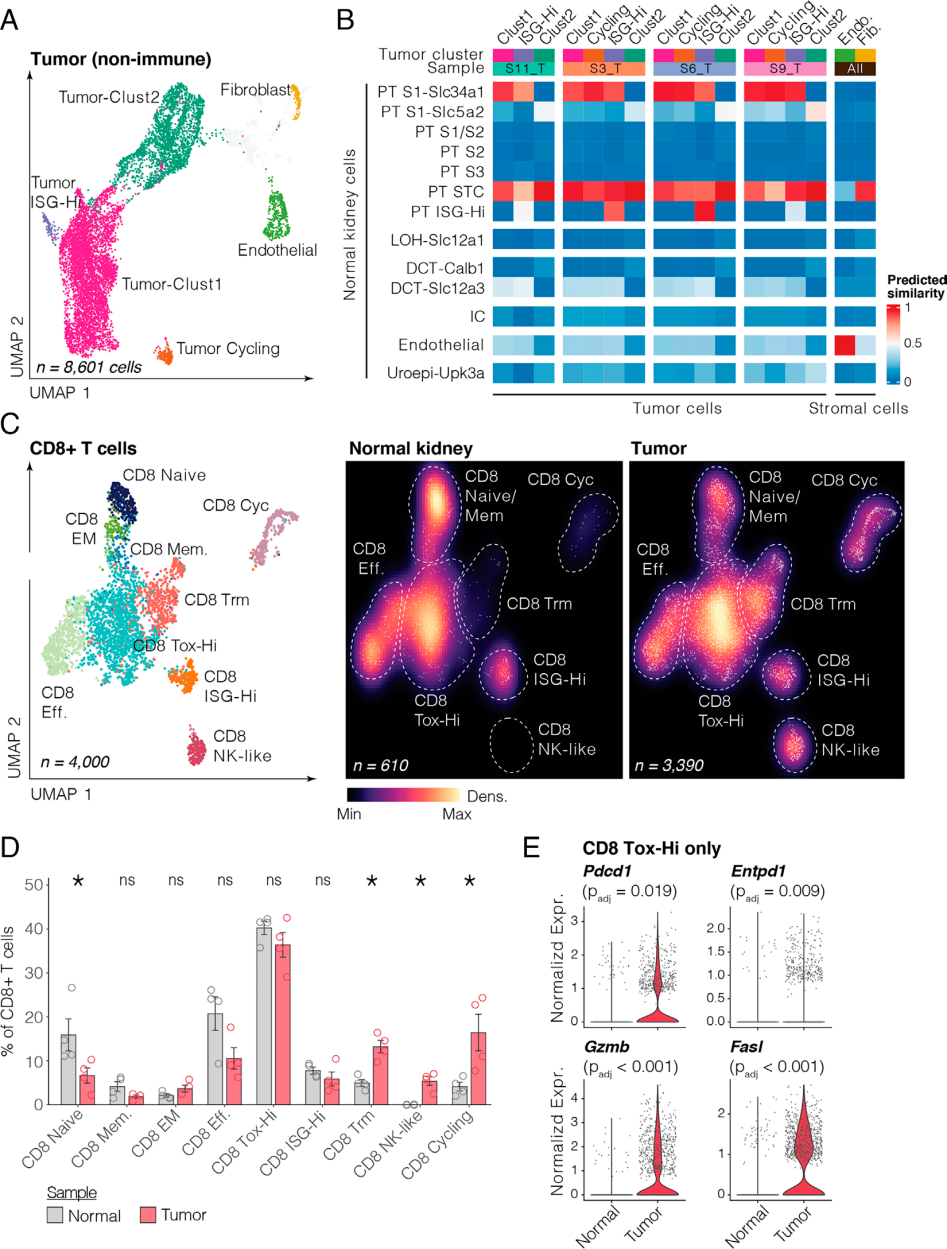
Single-cell RNA sequencing of Cre-less AAV model kidney tumors and normal mouse kidney tissue. (*A*) UMAP of nonimmune cells from 4 Cre-less AAV kidney tumors, colored and labeled by cluster/cell type. (*B*) Heatmap showing similarity of tumor cells (split by sample) and stromal cells to normal kidney cell types. Colors represent predicted similarity of a tumor or stromal subset in a given sample (columns) to normal cell types (rows). (*C*) *Left*, UMAP of CD8+ T cells from all four normal kidney and four tumor samples, colored and labeled by cluster/cell type. *Middle* and *Right*, UMAPs of CD8+ T cells split by normal kidney and tumor samples and colored by relative point density. (*D*) Quantification of CD8+ T cell subset proportions relative to total CD8+ T cells in normal kidney and tumor samples. *P*-values determined by the two-sided Wilcoxon rank-sum test. (*E*) Violin plots showing normalized expression of indicated genes in CD8 Tox-Hi cells from normal kidney and tumor samples. Bonferroni-adjusted *P*-values are from a logistic regression differential expression analysis using *Helicobacter* infection status as a covariate (*Materials and Methods*).

In humans, ccRCC is predicted to arise from a subset of proximal tubular epithelial cells expressing *VCAM1*, a hallmark of renal injury and kidney disease (55–57). To determine whether our Cre-less model tumors might derive from a similar cell of origin, we profiled 4 Cre-less AAV mouse tumors and 4 normal mouse kidneys using 10X 3′ single-cell RNA sequencing and compared the transcriptomes of tumor cells to those of normal microanatomical cell types spanning the nephron. Across normal kidney samples, 16,464 nonimmune cells were recovered, representing epithelial cells from the proximal tubule (PT), loop of Henle (LOH), distal convoluted tubule (DCT), collecting duct (IC and PC; intercalated and principal cells), Bowman’s capsule (podocytes and parietal epithelial cells), and uroepithelium, as well as fibroblasts and endothelial cells (*SI Appendix*, Fig. S8 *A* and *B* and Dataset S1 *A* and *B*). From tumor specimens, 8,601 nonimmune cells were recovered, including two major clusters of malignant cells, termed Tumor-Clust1 and Tumor-Clust2, with representation across all four samples. Tumor-Clust1 cells were enriched for a host of long noncoding RNAs, such as *Neat1*, *Malat1*, and *Snhg11*, some HIF targets, namely *Vegfa* and *Hk2*, and also *Ogt*, encoding O-GlcNAc transferase, a known positive regulator of HIF2 stability in ccRCC (58). Tumor-Clust2 cells showed higher expression of a different set of HIF targets, including *Ldha*, *Car9*, *Egln3*, and *Mif*, as well as the renal injury markers *Vcam1* and *Ptgds*, and EMT markers *Vim* and *Fn1*. We also identified two smaller subsets of malignant cells—one with high expression of interferon-stimulated genes *Ifit3* and *Cxcl10* (Tumor ISG-Hi) and another expressing *Mki67*, indicating active proliferation (Tumor Cycling)—in addition to tumor-associated fibroblasts and endothelium (Fig. 4*A*, *SI Appendix*, Fig. S8 *C*–*E*, and Dataset S1 *D*–*F*).

Next, we adapted a logistic regression approach previously used to identify the cell of origin of human ccRCC to determine the similarity between our tumor clusters and normal kidney cell types (55). We found that across samples, Tumor-Clust1, Tumor ISG-Hi, and Tumor Cycling cells, but not Tumor-Clust2 cells, showed high similarity to a subset of normal cells from the S1 segment of the convoluted PT with high expression of *Slc34a1*. Additionally, all tumor cells exhibited high similarity to a PT cluster in normal kidney expressing *Vcam1*, *Ptgds*, *Havcr1*, *Anxa3*, *Sox9*, and *Vim*, resembling scattered tubular cells (STC), a dedifferentiated and/or stem cell-like progenitor population for the proximal tubular epithelium (59–63) (Fig. 4*B*, *SI Appendix*, Fig. S8*F*, and Dataset S1*C*). The mapping of our tumor cells to PT STC recapitulates a key aspect of human ccRCC, wherein the proposed cell of origin is a *VCAM1*-high, *VIM*-high subset of PT cells (55, 57). However, in human ccRCC, in contrast to our mouse tumors, the cell of origin is also marked by *ITGB8* and *ALKP2*. We note that in healthy mouse kidney samples, in contrast to normal human kidney samples, *Itgb8* was expressed ubiquitously across all PT cells, and *Alpk2* showed near-zero expression across PT cells, suggesting a lack of conserved expression patterns between the mouse and human (*SI Appendix*, Fig. S8*F*). Thus, our model captures some, but not all characteristics of the cell of origin of human ccRCC.

### Characterizing the Immune Microenvironment of Cre-less AAV Model Tumors.

We next examined the 21,428 immune cells profiled across all eight single-cell samples to identify functional phenotypes enriched or depleted in Cre-less model tumors relative to normal mouse kidney. ccRCC is known to be a highly immune infiltrated lesion with a robust CD8+ T cell response (64–66). We found that our Cre-less model tumors exhibited a significantly higher proportion of tissue-resident CD8s (CD8 Trm), NK-like CD8s (CD8 NK-like), and proliferating CD8s (CD8 Cycling), as well as a lower proportion of naive CD8s (CD8 Naive), relative to normal mouse kidney (Fig. 4 *C* and *D*, *SI Appendix*, Fig. S9 *A*–*C*, and Dataset S2 *A* and *B*). Surprisingly, a cluster of CD8s expressing the master exhaustion transcription factor *Tox* was not proportionally enriched in tumor. However, within this CD8 Tox-Hi cluster, cells from Cre-less model tumors showed elevated expression of *Pdcd1/*PD-1, *Entpd1/*CD39 (a marker of terminal exhaustion) (67), and the cytotoxic effector molecules *Gzmb* and *Fasl*, suggesting chronic antigen exposure and active killing function consistent with an ongoing anti-tumor response (Fig. 4*E* and Dataset S2*C*).

Another hallmark of the ccRCC immune microenvironment is an extensive and suppressive myeloid infiltrate (66). We found that our Cre-less model tumors were significantly enriched for myeloid-derived suppressor cells (MDSC), proliferating MDSC (MDSC Cyc), and multiple macrophage subsets (Mac-Olfml3, Mac-Fabp5, Mac-*Cd163*, and Mac Cyc) relative to normal kidney (*SI Appendix*, Fig. S10 *A*–*D* and Dataset S2 *D* and *E*). Mac-*Cd163* cells in particular resemble canonical M2-like TAM, expressing *Cd163*, *Mrc1*, *Folr2*, and *Selenop* (68) (*SI Appendix*, Fig. S10*B*). In human ccRCC, these *CD163*+ macrophages increase in frequency with disease stage and play a major role in abrogating CD8+ T cell killing of tumors, contributing to worse prognosis (68). MDSC have also been observed in the tumor parenchyma of ccRCC patients and are associated with decreased overall survival (69, 70). These cells were almost completely absent in normal mouse kidney but represented on average 11.19% of all myeloid cells in Cre-less model tumor samples. Thus, Cre-less AAV tumors recapitulate a key aspect of ccRCC immunobiology, exhibiting enrichment of immunosuppressive myeloid subsets with known impact on disease progression and patient outcomes.

### Proof of Principle Efficacy Study of VEGF Targeted Therapy in the Cre-less AAV Model of ccRCC.

**Fig. 5.**
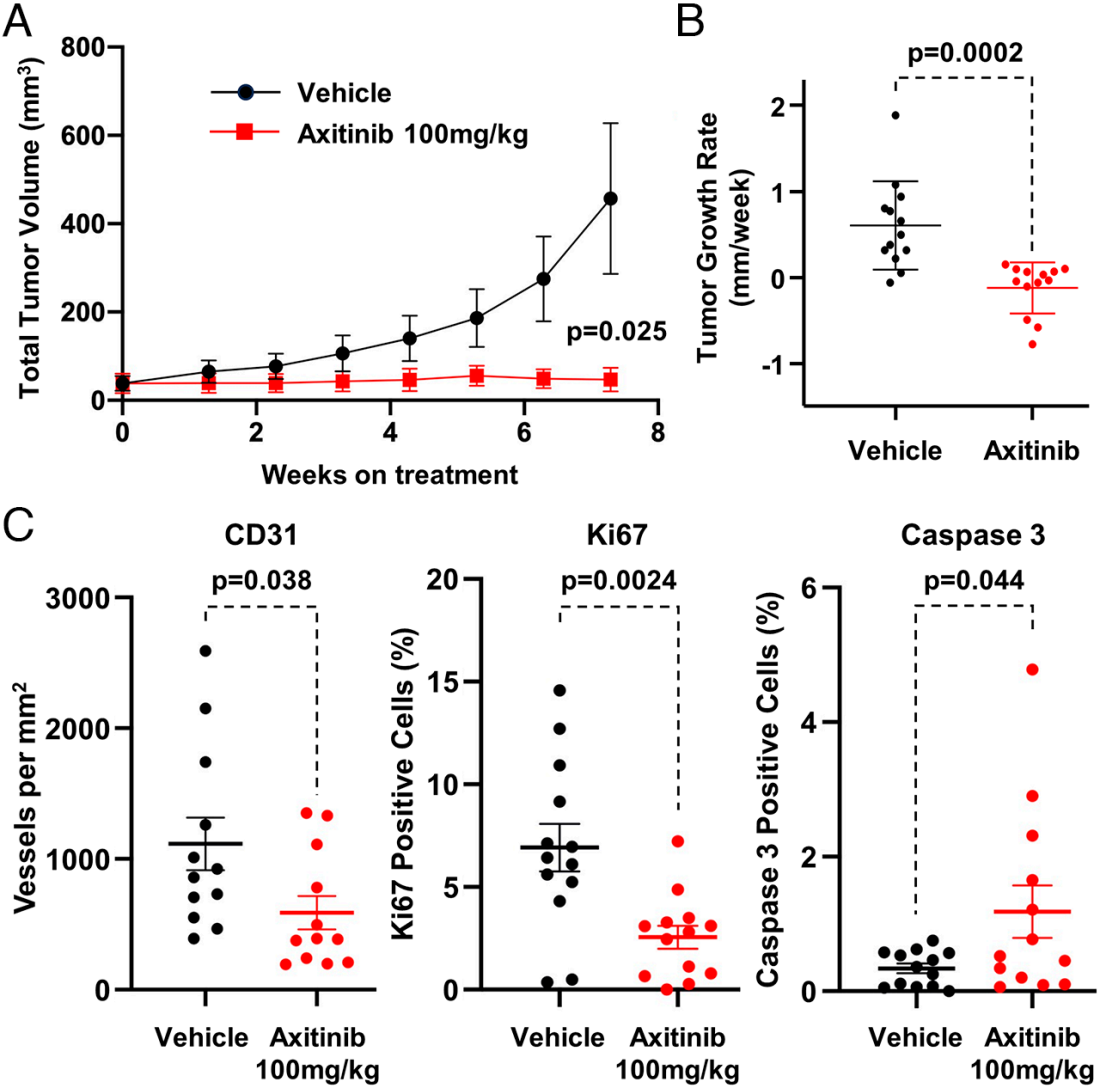
Potential of Cre-less AAV model for therapeutic studies. Axitinib study in Cre-less AAV model demonstrates response to VEGF pathway therapeutic intervention (100 mg/kg, b.i.d., p.o., 5 × week, 7-wk duration; n = 10 mice/treatment group). (*A*) Mean tumor volume, axitinib treated vs. vehicle. (*B*) Tumor growth rate, axitinib treated vs. vehicle. (*C*) Immunohistochemical staining of CD31, Ki67, and Caspase 3 in kidney tumors from Cre-less AAV model treated with axitinib (100 mg/kg) or vehicle. *P* value, unpaired *t* test, two-tailed.

VEGF antagonists are frequently the first-line therapy offered to patients with ccRCC and can produce both tumor regressions and delay tumor progression. To begin to explore the utility of our models for testing cancer therapies, we treated 10 mice with Cre-less AAV model tumors with axitinib (100 mg/kg, p.o., twice daily), which inhibits receptor tyrosine kinases including vascular endothelial growth factor receptor VEGFR-1, VEGFR-2, and VEGFR-3, and blocks neoangiogenesis. Compared to the vehicle control arm, axitinib significantly suppressed tumor growth rate (−0.1 mm diameter/week vs. +0.6 mm diameter/week, *P* = 0.0002; Fig. 5 *A* and *B*), resulting in stable disease over the 7-wk duration of the study. This was associated with a significant reduction in CD31 (microvessel density) and Ki67 (cell proliferation) immunostaining and an increase in caspase 3 (apoptosis) immunostaining (Fig. 5*C*).

### Evaluation of HIF-2α Inhibitor and Immune Checkpoint Inhibitors in Cre-less AAV Model of ccRCC.

In stark contrast, we did not see any effect on the growth of Cre-less AAV model ccRCCs after treatment with 1) anti-PD-1, 2) anti-CTLA4, 3) the HIF2 inhibitor PT2399, 4) anti-PD1 and PT2399, 5) anti-CTLA-4 and PT2399, 6) anti-PD1 and anti-CTLA4, or 7) anti-PD1, anti-CTLA4, and PT2399 compared to vehicle-treated mice (*SI Appendix*, Fig. S11 *A* and *B*) despite achieving adequate therapeutic blood levels of these agents (*SI Appendix*, Fig. S12 *A*, *C*, and *D*). Consistent with this, we did not see any changes in tumor-associated T cells, CD31, Ki-67, or in the HIF2-responsive gene product Cyclin D1 by immunohistochemistry of tumors removed at necropsy after 7 wk of therapy (*SI Appendix*, Fig. S13). The failure of PT2399 to modulate Cyclin D1 as well as other HIF targets was confirmed by qRT-PCR (*SI Appendix*, Fig. S7*B*) despite adequate drug accumulation in tumor tissue at high levels (*SI Appendix*, Fig. S12*B*). We conclude that the Cre-less model is HIF2 independent and insensitive to immune checkpoint blockade with currently approved agents. We were, however, able to demonstrate a robust tumor response to the mTOR inhibitor everolimus (5 mg/kg, daily, p.o.) in the Cre-less AAV model over a 5-wk period (366% vs. 74% relative tumor volume, *P* = 0.0174; *SI Appendix*, Fig. S14) that suggests that mTOR activation is driving tumor formation in this model as a result of *Tsc1* gene editing. The field therefore still awaits a faithful mouse model of human *VHL^−/−^* ccRCC.

While this work was being completed, two groups reported the use of CRISPR to generate mouse ccRCCs. Perelli and workers used *Pax8*-Cre mice crossed to mice harboring a L-S-L-Cas9 cassette in the *H11* locus to express Cas9 in the kidney and then injected those kidneys with AAVs containing sgRNAs against various TSGs (47). They generated tumors using AAVs that had two sgRNAs against *Cdkn2a/b*, one against *SetD2*, and an sgRNA against either *Nf2* or *Vhl*. Their analyses focused primarily on the *Nf2* mutated mice and neither the *Nf2* nor *Vhl* inactivated mice were interrogated with respect to HIF2 dependence. Rappold and coworkers electroporated an exposed mouse kidney to transiently express Cas9 and sgRNAs against *Vhl, Rb1*, and *Tp53* and to stably express *c-myc* (42). A single tumor was created that was used to generate multiple transplantable lines. These lines were shown to express Pax8 and HIF1α, but HIF2 status was not addressed.

The creation of mouse cancer models can be challenging for several reasons. First, it is clear that differences between mice and humans can dramatically affect the tissue- and cell type–specific susceptibility to cancer at the hands of bona fide human tumor suppressor genes and oncogenes. For example, germline *RB1* mutations predominantly cause retinoblastomas and sarcomas in man (71) and pituitary and thyroid cancers in mice (72, 73). Germline *TP53* mutations predominantly cause breast cancers, sarcomas, brain tumors, and adrenal gland tumors in man (74) and lymphomas in mice (75). Germline *APC* mutations cause large bowel tumors in man (76) and small bowel tumors in mice (77). Notably, germline *VHL* mutations do not appear to be sufficient to cause cancer in mice (78, 79). This could reflect the low probability of stochastically acquiring the cooperating mutations required to produce a VHL-associated neoplasm during a mouse lifetime. In this regard, it is perhaps notable that human chromosome 3p, which harbors multiple ccRCC tumor suppressor genes, is not syntenic to a single mouse chromosomal arm.

Complicating efforts to make a mouse *VHL^−/−^* ccRCC further, the kidney, including the functional unit referred to as the nephron, is made up of multiple cell types. The precise cell of origin for *VHL^−/−^* ccRCC has been debated for decades, although most studies point to a renal proximal tubular epithelial cell. A caveat is that *VHL* inactivation could theoretically lead to transdifferentiation, which could confound attempts to assign a true cell of origin, as well as other derangements in gene expression. This might explain the paradox that HIF2 expression is normally restricted to renal interstitial cells and endothelial cells, rather than the presumed cell of origin for ccRCC (proximal tubular epithelial cells). Careful examination of early renal lesions in *VHL* patients suggests that the appearance of HIF2 in such lesions occurs over time and coincides with worsening cellular atypia (10, 80). Finally, it seems likely that the chromosomal arms that are lost or gained in ccRCC harbor multiple ccRCC-relevant genes, including some that might act as haploinsufficient tumor suppressors (20, 53).

We used direct injections of AAVs into the kidney, coupled with either of two “kidney-specific” Cre drivers, to create murine ccRCCs. One of those drivers, PAX8, is clearly expressed in human *VHL*-defective ccRCCs and implicated in ccRCC pathogenesis (49, 51). Nonetheless, the tumors we produced were presumably derived from the cells that are the easiest to transform given the combinations of genetic manipulations we tested. It was already known that *Tsc2* mutations cause ccRCCs in rats (Ecker Rat) and mice (81). We presume, but have not proven, that these tumors are HIF1 dependent. Inactivation of *KEAP1* has been more tightly linked to murine and human papillary renal cell carcinomas than to ccRCCs (82–84). In future studies, we plan to exploit a mouse strain that we have designed and are currently validating in which Cre is driven by the *Epas1* locus to restrict Cre specifically to cells capable of expressing HIF2α. We will also test additional sgRNA combinations, including combinations that omit *Tsc1* and *Keap1* sgRNAs and include a *Hif1A* sgRNA, as well as technologies for deleting entire chromosomal regions. Finally, we might pursue species, such as rats and rhesus monkeys, whose genomes mimic the organization of human chromosome 3p.

## Materials and Methods

### Cell Culture.

3T3-J2 cells were obtained from Sigma-Aldrich and grown in DMEM supplemented with 10% FBS and 1% penicillin/streptomycin. 293FT cells were obtained from Thermo-Fisher Scientific and grown in DMEM as above.

### CRISPR Mouse Models.

The Pax8^tm1.1(Cre)mbu^/J transgenic mouse strain (common name Pax8 Cre/+) was generated by Meinrad Busslinger (48) and obtained from Jackson Labs (Strain No. 028196). Gt(ROSA)26Sor^tm1(CAG-cas9*, -EGFP)Fezh^/J mouse strain (common name Rosa26-LSL-Cas9-EGFP knockin) was generated by Feng Zhang (43) and obtained from Jackson Labs (Strain No.026175). Mice were maintained on a C57BL/6J background in standard housing.

#### Generation of the Ksp-Cre AAV model.

Rosa26-loxP-STOP-loxP(LSL)-Cas9/+ mice were orthotopically injected in the kidney with AAV expressing Cre recombinase under the *Cdh16(KSP)* promoter and sgRNAs for *Keap1, Pbrm1, Vhl, and Tsc1*.

#### Generation of the Cre-less AAV model.

LSL-Cas9/+ mice were crossed with Pax8 Cre mice to produce LSL-Cas9/+; Pax8 Cre/+ mice and orthotopically injected in the kidney with AAV expressing sgRNAs for *Keap1, Pbrm1, Vhl, and Tsc1.* All animal work was performed according to the guidelines of the Animal Care and Use Committee at the Frederick National Laboratory for Cancer Research and Dana-Farber Cancer Institute, Harvard Medical School.

### Orthotopic Mouse Kidney Injection of AAV.

Details of the surgical procedure for orthotopic mouse kidney injection of AAV are in *SI Appendix*, *Methods*.

### sgRNA Design.

Guide RNA sequences for *Vhl*, *Pbrm1, Keap1, Tsc1, Bap1, Cdkn2a, Hif1a, Setd2, Epas1*, and NT are in *SI Appendix*, *Methods*.

### AAV Plasmid Construction and Virus Production.

The AAV vector incorporates ITRs from AAV9, the human U6 promoter for sgRNA transcription, sgRNAs for genes of interest, and Cre recombinase under the *Cdh16 (Ksp)* promoter (Ksp-Cre AAV model). AAV vectors containing the sgRNAs were constructed using the Gateway system. For the pAAV-Gao-DEST construct, pX551 vector (Addgene #60957) was modified by inserting a Gateway cassette between XbaI and NotI. For pAAV-Gao-Ksp-Cre-DEST construct, a Ksp-Cre expression cassette was cloned into pAAV-Gao-DEST between PacI and KpnI.

For assembly of the sgRNA-containing pAAV-Gao-DEST construct, individual sgRNA and spacer fragments were amplified from pLentiCRISPRv2 plasmids containing the desired sgRNA using the KOD Xtreme PCR kit (Sigma) according to the manufacturer’s instructions. The pENTR223 backbone was similarly PCR amplified to yield a linear sequence. PCR products of the correct size were gel purified using the Qiagen gel extraction kit. Purified PCR products were combined by Gibson assembly (New England Biolabs) to yield pENTR223 containing the four sgRNAs of interest in the desired order. The resulting plasmid was amplified in NEB 5-alpha cells using spectinomycin selection and verified by Sanger sequencing. The verified sgRNA sequence was then inserted into AAV-Gao-DEST by the LR reaction. The LR reaction product was amplified in HB101 cells under ampicillin selection and verified by Sanger sequencing.

Large-scale AAV production was outsourced to Vigene Biosciences (Rockville, MD) (1 to 3 × 10^13^ GU/mL) and stored at −80 °C.

### RNA Isolation and qPCR.

RNA was extracted from frozen mouse tumor and normal kidney tissue with the RNeasy mini kit (Qiagen) according to the manufacturer’s instructions and converted to cDNA with AffinityScript QPCR cDNA Synthesis Kit (Agilent). Gene expression was measured by Real-Time PCR using LightCycler 480 SYBR Green I on a LightCycler 480 Real-Time PCR system (Roche) per the manufacturer’s instructions. All assays were run in triplicate and gene expression was calculated as comparative CT (ΔΔCT) values. Gene expression was evaluated for the following genes: *Actb, Ccnd1, Vegfa, Slc2a1, Ndrg1, Stat1, Pdk1, Pgk1, Epas1, Car9*, and *Hif1a* (primer sequences in *SI Appendix*, *Methods*).

### Determination of In Vitro and In Vivo CRISPR/Cas9 Gene Editing Efficiency.

Genomic DNA was purified from mouse tissues on the Maxwell 16 Instrument (Promega) using the Maxwell 16 Tissue DNA Purification Kit according to the manufacturer’s instructions. PCR amplification of each gene from the 3T3-J2 cell, mouse tumor, or normal kidney genomic DNA was performed using a NEST primer set that flanks the sgRNA site (*SI Appendix*, *Methods*). NEST PCR products were gel purified and subjected to a second PCR amplification using the internal SEQ primer set. The purified SEQ PCR amplicons were pooled and sequenced on the Illumina MiSeq system using TruSeq DNA Nano Libraries and paired-end sequencing. All samples had above 85% of Q30 bases and yields between 395 and 573 thousand pass filter reads. Samples were trimmed for adapters using Cutadapt and the trimmed reads were analyzed with CRISPResso2, a software pipeline designed to enable rapid and intuitive interpretation of genome editing experiments (85).

### In Vivo Tumor Development and Monitoring.

Mouse tumor development was initiated by orthotopic kidney injection of AAV expressing sgRNAs for *Vhl, Pbrm1, Keap1*, and *Tsc1* (up to 3 × 10^11^ GU/mouse) following the surgical procedure as described in *SI Appendix*, *Methods*. Tumor development was monitored by serial MRI. Mice were killed by CO_2_ asphyxiation at humane end points per institutional ACUC guidelines (20 mm maximum dimension) for tumor collection and biochemical analysis.

### MRI.

Early detection and monitoring of tumors was achieved by 3D MRI utilizing a 3.0 T MRI clinical scanner (Philips Intera Achieva, Best, The Netherlands) using a custom-built multimouse volume receive array coil (Lambda Z Technologies, Baltimore, MD) for high-throughput imaging of three mice simultaneously. Noncontrast nongated T2w MRI was initiated 8 to 10 wk after AAV injection then serial imaging was performed to monitor growth (biweekly) and response to therapy (weekly). Details of the MRI protocol are in *SI Appendix*, *Methods*. ImageJ version 1.53 (86) was used to visualize the MRI tumor images. Maximum diameter measurements were taken from the image in the coronal plane to evaluate tumor growth and response to therapy.

### RNA Sequencing and Analysis.

Ten mouse kidney tumors and ten normal kidneys were collected from an initial cohort of 10 Cre-less model mice specifically produced for RNA sequencing and genomic analysis. Separate aliquots were used to produce RNA for RNAseq and DNA for whole-genome sequencing (WGS) and copy number analysis. RNAseq was performed by Novogene (Novogene Corporation, Inc., CA), and details are presented in *SI Appendix*, *Methods*. The raw data have been uploaded to the Gene Expression Omnibus (https://www.ncbi.nlm.nih.gov/geo/) with accession number GSE275231.

### Whole-Genome Sequencing and Copy Number Analysis.

The TruSeq Nano DNA Library prep kit (Illumina, 15041110 D) was used to prepare samples for whole-genome sequencing. The methodologies for WGS and variant/copy number analysis are detailed in *SI Appendix*, *Methods*. The average sequencing depth was between 53X and 69X mean genome coverage for tumor samples and normal samples. Details are in *SI Appendix*, Table S3 and *Sequencing Statistics*.

### Single-Cell Sequencing.

Mouse tumor and normal kidney samples (~50 mg per unit dissociation mix) were dissociated into single-cell suspensions, and libraries were generated utilizing the Chromium Next GEM Single Cell v 3.1 platform (10X Genomics) and sequenced across two flowcells-one P2 flowcell on a NextSeq 2000 system and one S2 flowcell on a NovaSeq 6000 system (Illumina). Raw data have been uploaded to the Gene Expression Omnibus with accession number GSE275339. Details are in *SI Appendix*, *Methods*.

### scRNA-Seq Data Preprocessing.

Preprocessing of scRNA-Seq data, including alignment, read counting, ambient RNA decontamination, and multiplet removal, was performed using field standard tools and parameters (*SI Appendix*, *Methods*).

### Normal Kidney and Tumor Cell Type Annotation.

Annotation of normal kidney and tumor cells was performed with iterative rounds of dimension reduction, integration, clustering, and differential expression analysis. Removal of normal kidney and tumor cells driven by residual ambient RNA contamination was performed as described in *SI Appendix*, *Methods*.

### Immune Cell Annotation and Analysis.

Annotation of immune cells from normal kidney and tumor samples was performed with iterative rounds of dimension reduction, integration, clustering, and differential expression analysis (*SI Appendix*, *Methods*).

### Cell of Origin Analysis.

To determine the cell of origin of model tumor cells, we adapted an approach previously used to identify the cell of origin of human ccRCC by comparing transcriptional profiles of RCC tumor cells with healthy kidney tissue (*SI Appendix*, *Methods*).

### In Vivo Drug Studies.

When tumors reached ~24 to 35 mm^3^, mice were randomized by tumor volume into treatment groups, dosed with drug or vehicle according to study design, and monitored for tumor response by weekly MRI. Body weights were taken twice weekly. At study end point, mice were euthanized humanely by CO_2_ asphyxiation and tumors were removed for biochemical analysis. A separate group of five mice per treatment arm were treated for 4 d and then euthanized for PT2399 tissue analysis and evaluation of HIF-α and HIF target gene expression.

#### Axitinib study.

Treatment arms (10 mice per arm) included axitinib, 100 mg/kg in 0.5% carboxymethyl cellulose, p.o., b.i.d.,5 × week and vehicle dosed per treatment arm for 7 wk.

#### PT2399 and immunotherapy study.

Treatment arms (10 mice per arm) included 1) anti-murine-PD1 (muDX400, Merck & Co., Inc., Rahway, NJ), 10 mg/kg, i.p., Q5 for five doses; 2) anti-murine CTLA4 (Catalogue # BP0164, clone 9D9, BioXCell), 5 mg/kg, i.p., Q5 for five doses; 3) PT2399, 30 mg/kg in 10% EtOH, 30% PEG400, 60% MCT(0.5% methylcellulose/0.5% Tween 80), p.o., b.i.d., 5 × week; 4) anti-murine PD1 and PT2399; 5) anti-murine CTLA-4 and PT2399; 6) anti-murine-PD1 and anti-murine CTLA4; 7) anti-murine PD1, anti-murine CTLA4, and PT2399; and 8) vehicles dosed per treatment arms for 7 wk.

#### Everolimus study.

Treatment arms (five mice per arm) included everolimus, 5 mg/kg in 10% DMSO, 40% PEG300, 5% Tween 80, 45% saline, p.o., once daily, 5 × week, and vehicle dosed per treatment arm for 5 wk.

### Determination of PT2399, Anti-PD-1, and Anti-CTLA4 in Animal Tissues.

#### Determination of murine anti-PD-1 (muDX400) and anti-CTLA4 in mouse serum.

Levels of therapeutic antibodies were evaluated by a bioanalytical method using the MSD electrochemiluminescence (ECL) format as described in *SI Appendix*, *Methods*.

#### Determination of PT2399 in mouse blood and tumor.

Mouse biological samples (blood and tumor homogenate) were prepared with protein precipitation and analyzed with LC–MS/MS as described in *SI Appendix*, *Methods*.

### Immunohistochemistry of Tumor Tissues.

Immunohistochemical staining of tumors to assess gene editing for Pbrm1, Tsc1 (pS6) and Vhl (Ca9), and Pax8 and GFP was performed as described in *SI Appendix*, *Methods*.

### Statistical Analysis.

Kaplan–Meier survival analysis and statistical analysis were performed using GraphPad Prism Version 8.4.3 software. scRNA-Seq analysis was performed using R version 4.1. *P* values < 0.05 are considered statistically significant.

## Supplementary Material

Appendix 01 (PDF)

Dataset S01 (XLSX)

Dataset S02 (XLSX)

Dataset S03 (XLSX)

Dataset S04 (XLSX)

## Data Availability

Source code for scRNA-Seq analysis is available on GitHub at https://github.com/kevinbi2599/ccRCC_Creless_Model_scRNA (87). Additional data related to this paper may be requested from the authors. All plasmids are available from the authors. RNA sequencing raw data files and single cell sequencing raw data files have been deposited in the Gene Expression Omnibus (https://www.ncbi.nlm.nih.gov/geo/) [RNA sequencing data: GSE275231 (88); Single cell sequencing data: GSE275339 (89)]. All other data are included in the manuscript and/or supporting information.
