# Supplementary material for: Toward a CRISPR-based mouse model of Vhl-deficient clear cell kidney cancer: Initial experience and lessons learned
Source: Proc Natl Acad Sci U S A. 2024 Oct 4;121(41):e2408549121. doi: 10.1073/pnas.2408549121 (PMC11474080; doi:10.1073/pnas.2408549121)
Supplement: Supplementary file 1 — Appendix 01 (PDF) [file pnas.2408549121.sapp.pdf]

Supporting Information for

Title: Towards a CRISPR-Based Mouse Model of *Vhl*-deficient Clear Cell Kidney Cancer: Initial Experience and Lessons Learned

Authors: Laura A. Stransky[a,#], Wenhua Gao[a,#], Laura S. Schmidt[c,d,#], Kevin Bi[e], Christopher J. Ricketts[c], Vijyendra Ramesh[a], Amy James[f], Simone Difilippantonio[f], Lilia Ileva[g], Joseph D. Kalen[g], Baktiar Karim[h], Albert Jeon[h], Tamara Morgan[h], Andrew C. Warner[h] , Sevilay Turan[i], Joanne Unite[i], Bao Tran[i], Sulbha Choudhari[j], Yongmei Zhao[j], Douglas E. Linn[k], Changhong Yun[l], Sripriya Dhandapani[m], Vaishali Parab[m], Elaine M. Pinheiro[n], Nicole Morris[h], Lixia He[a], Sean M. Vigeant[a], Jean-Christophe Pignon[o], Maura Sticco-Ivins[o], Sabina Signoretti[o], Eliezer M. Van Allen[e], W. Marston Linehan[c], William G. Kaelin, Jr.[a,b,*]

Affiliations:

[a] Department of Medical Oncology, Dana-Farber Cancer Institute, Harvard Medical School, Boston, MA 02215.

[b] HHMI, Chevy Chase, MD 20815

[c] Urologic Oncology Branch, Center for Cancer Research, National Cancer Institute, NIH, Bethesda, MD 20892

[d] Basic Science Program, Frederick National Laboratory for Cancer Research, Frederick, MD 21702

[e] Department of Medical Oncology, Dana-Farber Cancer Institute, Boston, MA 02115, USA; Center for Cancer Genomics, Dana-Farber Cancer Institute, Boston, MA 02115, USA; Broad Institute of Harvard and MIT, Cambridge, MA 02142, USA.

[f] Animal Research Technical Support, Frederick National Laboratory for Cancer Research, Frederick, MD 21702

[g] Small Animal Imaging Program, Frederck National Laboratory for Cancer Research, Frederick, MD 21702

[h] Molecular Histopathology Laboratory, Frederick National Laboratory for Cancer Research, Frederick, MD 21702, USA

[i] NCI CCR Sequencing Facility, Frederick National Laboratory for Cancer Research, Frederick, MD, USA.

[j] Advanced Biomedical and Computational Science, Frederick National Laboratory for Cancer Research, Frederick, MD, USA.

[k] Quantitative Biosciences, Merck & Co., Inc., Boston, MA, USA

[l] Pharmacokinetics, Merck & Co., Inc., Boston, MA, USA

[m] Pharmacokinetics, Merck & Co., Inc., South San Francisco, CA, USA

[n] Discovery Oncology, Merck & Co., Inc., Boston, MA, USA

[o] Harvard Medical School, Boston, MA 02115, USA; Department of Pathology, Brigham and Women's Hospital, Boston, MA 02115, USA; Department of Oncologic Pathology, Dana-Farber Cancer Institute, Boston, MA 02115, USA.
[#]These authors contributed equally.
*To whom correspondence may be addressed. Email:william_kaelin@dfci.harvard.edu

This PDF file includes:

Materials and Methods
Figures S1 to S15
Tables S1-S3
SI References

Other supporting materials for this manuscript include the following:

Dataset S1. Cell-level metadata and differential expression results for non-immune cells from normal kidney and tumor scRNA-Seq

Dataset S2. Cell-level metadata and differential expression results for immune cells from normal kidney and tumor scRNA-Seq

Dataset S3. Gene count and quality control data for RNAseq derived from 10 mouse tumors and 10 normal mouse kidney samples

Dataset S4. Ambient RNA signatures and GSEA results for normal kidney and tumor scRNA-Seq

Materials and Methods

sgRNA design

sgRNA for Vhl: TACCCGATCTTACCACCG
sgRNA for Pbrm1: TCAGGACGGCTCATCAGT
sgRNA for Keap1: CGCAGGACGGTAACCGAAC
sgRNA for Tsc1: CGAGGGGGCTTTGACTCT
sgRNA for Bap1: CGCTTAGCAAGGCGTGA
sgRNA for Cdkn2a:CGGTGCAGATTCGAACTGC
sgrna for Hif1a:TGCACCCTAACAAGCCG
sgRNA for Setd2: CAGCCGCCACCGAAGAT
sgRNA for Epas1: TGAAGAAGTCACGCTCG
sgRNA for NT control:GAGGCTAAGCGTCGCAA

RNA isolation and qPCR

RNA was extracted from frozen mouse tumor and normal kidney tissue with the RNeasy mini kit (Qiagen) according to manufacturer's instructions and converted to cDNA with AffinityScript QPCR cDNA Synthesis Kit (Agilent). Gene expression was measured by Real-Time PCR using LightCycler 480 SYBR Green I on a LightCycler 480 Real-Time PCR system (Roche) per manufacturer's instructions. All assays were run in triplicate and gene expression was calculated as comparative CT (ΔΔCT) values. Gene expression was evaluated for the following genes using the listed primer sets:

Actb: forward 5'- GTGACGTTGACATCCGTAAAGA;
reverse 5'- GCCGGACTCATCGTACTCC
Ccnd1: forward 5'-GCAGAAGGAGATTGTGCCATCC;
reverse 5'-AGGAAGCGGTCCAGGTAGTTCA
Vegfa: forward 5'-CTGCTGTAACGATGAAGCCCTG;
reverse 5'-GCTGTAGGAAGCTCATCTCTCC
Slc2a1 forward 5'- GCTTCTCCAACTGGACCTCAAAC;
reverse 5'- ACGAGGAGCACCGTGAAGATGA
Ndrg1 forward 5'- TTGCTGTCTGCCATGTGGATGC;
reverse 5'- GACTCCAGGAAGCATTTCAGCC
Stat1 forward 5'- GCCTCTCATTGTCACCGAAGAAC;
reverse 5'- TGGCTGACGTTGGAGATCACCA
Pdk1 forward 5'- CTACCAGCCATGTCAGAGGATG;
reverse 5'- AGGCTGGTTTCCACCGTAGACA
Pgk1 forward 5'- GATGCTTTCCGAGCCTCACTGT;

reverse 5'- ACCAGCCTTCTGTGGCAGATTC
Epas1 forward 5'- GGACAGCAAGACTTTCCTGAGC;
reverse 5'- GGTAGAACTCATAGGCAGAGCG
Car9 forward 5'- GGCGAACGATTGAGGCTTCCTT;
reverse 5'- GCTGGTGACAGCAAAGAGAAGG
Hif1a forward 5'- CCTGCACTGAATCAAGAGGTTGC;
reverse 5'- CCATCAGAAGGACTTGCTGGCT

Orthotopic mouse kidney injection of AAV

Mice (M/F, 8-12 weeks of age) were anesthetized with Isoflurane to effect. After the mice were fully anesthetized, the surgical field was sterilized and the skin was retracted with forceps. Sterile scissors were then used to make a ~ 2 cm incision along back of animal. After kidney was exposed, up to 30 µl of concentrated adeno-associated virus (AAV) (titer, up to 3 x 10^{11} genome copy/mouse) was injected near the lower kidney pole into the renal parenchyma. The surgical wound was closed in two layers- the abdominal wall with sutures and skin with autoclips. The mice were placed on a heat source (heat-pack or heating pad) after injection to prevent hypothermia. Animals received 0.05 mg/kg buprenorphine SQ immediately after the procedure. The autoclips were removed 7-10 days later.

Determination of *in vitro* and *in vivo* CRISPR/Cas9 gene editing efficiency

Vhl NEST primer set: forward 5'-AA AGA AAA GGA AAG GAA ACA AAC AGA AAC; reverse 5'-GCC TAG CGC AGC AAT TAT GAC ACA
Vhl SEQ primer set: forward 5'-CGG TGC TGC GCT CGG TGA ACT CG; reverse 5'-CGG GGT AGA TGC AGT GGG TAG GGA CAA GAT
Pbrm1 NEST primer set: forward 5'-TGA CTC CCT CTT CTG GAG TGT; reverse 5'-CTG CTG AGG CCA TGA AGA GTA
Pbrm1 SEQ primer set: forward 5'-TGC CGT CTA GTC TTC TCC AGG; reverse 5'-CAC CAC TAC CCA GCA ACA CTG
Keap1 NEST primer set: forward 5'-CCC TGA AGG CCC GCG AGT TGA GA; reverse 5'-AGG CTC GAA CCA CGC TGT CAA TC
Keap1 SEQ primer set: forward 5'-CCG CAG CAG CCA GTT CCT GCC CC; reverse 5'-TCA TAT TTG ACC TGC AGG GTC ACG

Magnetic resonance imaging

Early detection and monitoring of tumors was achieved by magnetic resonance imaging (MRI) utilizing a 3.0T MRI clinical scanner (Philips Intera Achieva, Best, The Netherlands) using a custom-built multi-mouse volume receive array coil (Lambda Z Technologies, Baltimore, MD) for high throughput imaging of three mice simultaneously. Non-contrast non-gated T2w MRI was

initiated 8-10 weeks after AAV injection; then weekly serial imaging was performed to monitor growth and response to therapy.

The animal body temperature (thermostat controlled heated table at 34–37 °C) was maintained from the time the animal entered the imaging room, through anesthesia induction, imaging, and until recovery from anesthesia. Isoflurane anesthesia was administered via an induction chamber (3% pre-imaging) and nose cone (1.5–2% during imaging) with oxygen as the carrier gas at a flow of 1 L/min. Pulmonary function was monitored (MP150, Biopac Systems, Inc, Goleta, CA) during scanning and the anesthesia (1.5–2% isoflurane) was regulated to maintain a pulmonary rate of 40 breaths per min (bpm) to reduce motion artifacts.

An initial survey scan with slices in the three orthogonal directions (sagittal, coronal, and axial) were applied to locate mouse kidneys and determine the imaging planes. Multislice T2 weighted images were acquired using a turbo spin echo sequence (T2w-TSE) applied in coronal view with the following parameters: field of view 160 mm x 40 mm; in-plane resolution 0.18 mm × 0.18 mm; slice thickness 0.5 mm; repetition time (TR) 4570 ms; echo time (TE) 45 ms. A Spectral Presaturation with Inversion Recovery (SPIR) sequence (Philips Healthcare, Brest, The Netherlands) was used to suppress the signal from adipose tissue and improve the contrast between cyst, tumors and fat tissue surrounding the kidneys.

RNA sequencing and analysis

Mouse tumors and normal kidneys were flash frozen and cryo-pulverized in liquid nitrogen for RNA extraction for RNA sequencing. The resultant powder was resuspended in 1 mL of Trizol Reagent (Invitrogen, CA, USA) and total RNA extraction was performed in accordance with the manufacturer's protocol. RNA concentrations and purities were evaluated using an Agilent 4150 TapeStation System (Agilent Technologies, Inc., CA, USA) by the NCI CCR Genomics Core (Bethesda, MD) and RNA was stored at -80°C. RNAseq library preparation and transcriptome sequencing was performed by Novogene (Novogene Corporation, Inc.,CA, USA) using Illumina PE150 technology (Illumina, Inc., CA, USA). The gene count data provided by Novogene were analyzed by the NIH Integrated Data Analysis Portal (NIDAP) using the RNA sequencing data analysis pipeline to produce PCA plots, gene clustering heatmaps, and differential gene expression profiles, and perform pathway/gene set enrichment analysis. The raw gene count data and the quality control data are presented in Dataset S3. The percentage of gene editing within the tumor samples for the 4 targeted genes is also shown based on WGS data (Dataset S3).

Whole Genome Sequencing and Copy Number Analysis

The TruSeq Nano DNA Library prep kit (Illumina, 15041110 D) was used to prepare samples for whole genome sequencing. The whole genome libraries were pooled together and sequenced on a NovaSeq 6000 instrument at 2x150bp read length using the S4 configuration. Sequencing was performed following the manufacturer's instructions. The sequencing run was demultiplexed using Illumina Bcl2fastq v2.20 software tool. The raw fastq files were used as input to run DRAGEN 4.0.5 (Illumina, USA) for mapping and variant calling. The mouse reference genome mm10 (GRCm38) was used as reference. DRAGEN was run for each tumor with six pooled normal samples and subsampled to 100x as pairs using the somatic variant calling mode to call the structural variants and SNVs. For normal samples, the germline joint genotyping was used to call variants. For Copy Number Variation, analysis was performed using DRAGEN v 4.0.5 CNV pipeline and self-normalization mode with germline join genotyping variants for combined normal samples.

Single cell sequencing sample prep, generation of libraries and sequencing

Mouse tumor and normal kidney samples (~50 mg per unit dissociation mix) were dissociated into single cell suspensions using a cocktail of collagenase (VitaCyte 001-2030 Collagenase MA, 13units/µL) and protease (VitaCyte 003-1000 BP Protease, 0.88 units/µL) in RPMI media at 37 C for 5 min. with pipetting to break up tissue as needed. The digestion mix was passed through a 70 µM mesh followed by a 40 µM mesh and quenched with cold PBS/10% FBS. The flow-through was centrifuged to remove the enzymes and quenching solution. Red blood cells (RBCs) were lysed with cold ACK buffer for 3 min. at room temperature. The single cell suspension was washed and counted using a hemocytometer-Trypan Blue. Single-cell RNA-seq libraries were generated utilizing the Chromium Next GEM Single Cell v 3.1 platform (10X Genomics, US). Single-cell suspensions were prepared by diluting cells to a concentration of 1000 cells/µL in 1x PBS with 0.04% BSA. Subsequently, these cells were integrated into a real-time polymerase chain reaction (RT-PCR) master mix with the aim of targeting approximately 10,000 cells. Following the manufacturer's instructions, this mixture was loaded into a Next GEM Chip G, along with Single Cell v3.1 gel beads and partitioning oil. The cDNA molecules underwent amplification, dual indexing, and pooling, as per the manufacturer's guidelines (10X Genomics, US) and sequencing libraries were quantified using the Agilent High Sensitivity ScreenTape. To ensure comprehensive sequencing, all single-cell libraries were subjected to a customized paired-end format with dual indexing (28/10/90-bp for v3.1 libraries), following the recommendations of 10X Genomics. Finally, these libraries were sequenced across two flowcells-one P2 flowcell on a NextSeq 2000 system, and one S2 flowcell on a NovaSeq 6000 system (Illumina, US).

scRNA-seq data preprocessing

Demultiplexing of sequencing results, barcode processing, read alignment, and UMI counting were performed using the 10x Genomics CellRanger pipeline (v6.1.2) with the include_introns parameter set to “false”. Reads were aligned to the mm10-2020-A reference genome. After exclusion of low RNA content droplets, confidently mapped, non-PCR duplicate reads were counted to generate a gene-barcode matrix for each sample.

To abrogate sample-to-sample technical differences arising from cell-free RNA profiles, an ambient RNA decontamination step was performed on each sample individually using CellBender (v0.2.0). Briefly, raw counts for each sample were provided as input to the remove-background module of CellBender (3). Remove-background was run on all samples using the “full” model, 150 training epochs, a target false positive rate of 0.01, an empty drop training fraction of 0.5, and 40,000 total droplets included. For each sample, the expected cells parameter was derived from the estimate outputted by CellRanger.

To exclude data from droplets encapsulating more than one cell, we performed multiplet detection and removal on CellBender-cleaned counts matrices using the Scrublet package (Python, v0.2.1) (4). For each sample, an expected doublet rate parameter of 0.1 was used, and doublet score thresholds were tuned manually to partition putative singlet and neotypic doublet modes in the score distribution. Predicted doublets were then removed from gene-barcode matrices, yielding a singlet-only, ambient RNA-subtracted counts matrix for each sample. All further quality control, feature selection, dimension reduction, unsupervised clustering, and differential expression analyses were performed using the Seurat R package (v4.2.0) (5).

Normal kidney and tumor cell type annotation

Prior to clustering and cell type annotation, the 8 samples were merged into a single Seurat object. Cells with fewer than 200 genes detected or 20% or more of counts attributed to mitochonodrially-encoded transcripts were removed. Genes detected in fewer than three cells across samples were also excluded. Raw counts were then normalized and scaled using the default Seurat log-normalization approach.

For an initial overview of the data, we performed principal components analysis (PCA) on the normalized expression matrix using the 3000 most highly variable genes in the dataset. The first 30 principal components (PCs) were then used to perform Louvain clustering and uniform manifold approximation and projection (UMAP) for visualization in two dimensions. Clusters were then broadly defined as immune or non-immune based on a per-cluster cutoff of 25% percent of cells expressing *Ptprc*, encoding CD45.

To annotate normal nephron cell types, we re-normalized and re-scaled cells from normal kidney samples, excluding cells from broadly defined immune clusters. For this subset of the data, we again performed PCA using 3000 highly variable genes, and additionally integrated the first 30 PCs using the R package Harmony (v0.1.0) (6) to correct for variation based on animal sex, as mice are known to exhibit highly sexually dimorphic transcriptional programs across proximal tubule segments (7). The 30 Harmony-corrected dimensions were then used for Louvain clustering and UMAP. We performed differential expression analysis comparing cells of each Louvain cluster to all other cells in the subset using a logistic regression framework ("LR" method implemented by Seurat) with Bonferonni correction for multiple hypothesis testing and animal sex included as a latent variable. Clusters were annotated as major nephron cell types based on significant differential expression of known marker genes (2, 7-10).

Despite ambient RNA decontamination using CellBender, we observed substantial variation in non-immune normal kidney cells driven by residual cell-free RNA profiles. In particular, we identified two major groupings of proximal tubule cells, termed PT_A and PT_B, both spanning the S1 and S2 segments (SI Appendix, Fig. S15A). PT_A cells, relative to PT_B, exhibited significantly higher expression of genes commonly observed in ambient RNA profiles, including *Ftl1*, *Fth1*, and genes encoding ribosomal subunits, and also exhibited a lower number of total genes and total counts per cell, suggesting lower overall library quality (SI Appendix, Fig. S15B). To quantitatively determine whether PT_A cells showed signs of greater cell-free RNA contamination, we used the raw, non-background-corrected expression matrices from each normal kidney sample to define gene signatures describing droplets with low RNA content; for each sample, we identified the top 200 most highly expressed genes within droplets with 50-200 total counts and determined the intersect and union of these sets. We then performed GSEA (fgsea R package, v1.18.0) comparing PT_A and PT_B cells in the scope of these signatures, and found that PT_A cells were significantly enriched for cell-free RNA gene sets (SI Appendix, Fig. S15C , and Dataset S4A-B). We therefore chose to omit these cells from further analysis. Following removal of PT_A, we iteratively performed re-normalization and re-scaling, PCA, Harmony correction, Louvain clustering, UMAP, and differential expression to identify cell types and states with distinct expression profiles, resulting in 16,446 annotated normal kidney cells for the cell of origin analysis.

To annotate tumor and tumor-associated stromal cells, we re-normalized and re-scaled cells from Cre-less AAV tumor samples, excluding cells from broadly defined immune clusters, and again performed PCA, Harmony integration, Louvain clustering, UMAP, and differential expression analysis. We initially identified three main tumor clusters, termed Tumor_Neat1, Tumor_Cpe, and Tumor_Ftl1 (SI Appendix, Fig. S15D). Tumor_Ftl1 cells, but not Tumor_Cpe, displayed substantially lower genes and counts per cell compared to Tumor_Neat1 (SI Appendix,

Fig. S15E). Tumor_Cpe and Tumor_Ftl1 both showed enrichment of cell-free RNA gene sets derived from the 4 tumor samples relative to Tumor_Neat1, and Tumor_Ftl1 was enriched for these gene sets relative to Tumor_Cpe (SI Appendix, Fig. S15F and Dataset4C-F). Additionally, compared to Tumor_Neat1, Tumor_Cpe were significantly enriched for a host of HIF target genes, renal injury-associated genes, and EMT genes, suggesting a true distinct cell state. Thus, we chose to remove Tumor_Ftl1 from further analysis, but retain Tumor_Cpe. Final iterations of subsetting, re-clustering, and re-annotation yielded 8,601 annotated tumor and stromal cells for subsequent analysis.

Immune cell annotation and analysis

To annotate immune subsets, we performed iterations of subsetting, re-clustering, and re-annotation via differential expression as described above, beginning with broadly identified immune cells from normal kidney and tumor samples. Granular cell type and state annotations were achieved via subsetting of CD8+ T cells, CD4+ T cells, NK cells, and myeloid cells into discrete objects. For all Harmony integration in immune cells, we adjusted for sample as the primary source of variation. For all differential expression analyses between clusters in immune cells, we used a logistic regression framework with Bonferonni correction for multiple hypothesis testing and sample as the sole latent variable.

During the course of the study, a colony breakout of *Helicobacter hepaticus* developed and 2/4 tumor-bearing mice used in the single cell sequencing study became infected. Mice were quarantined until tumors reached appropriate size for harvest and did not display any health issues. In comparing immune cells from tumor and normal samples, we applied multiple measures for ruling out *Helicobacter hepaticus* infection as a driver of phenotypes of interest. For differential expression analysis between CD8 Tox-Hi cells from tumor and normal samples, *Helicobacter* infection status was used as the sole latent variable. When comparing CD8+ T cell and myeloid cell frequencies between tumor and normal specimens, we quantified cell type proportions on a sample to sample basis and found that *Helicobacter* infection status was not a driving factor behind group-level differences.

Cell of origin analysis

To quantify the similarity of model tumor cells to normal kidney cell types, we adapted a logistic regression approach used to identify the cell of origin of ccRCC in human scRNA-Seq data (1). For each normal kidney cluster, we fit a one-versus-rest binomial logistic regression model with elastic net regularization using all nephron cell types, normal kidney fibroblasts, and normal kidney endothelial cells as a training set. Prior to model training, we added a feature selection step; differential gene expression analysis was performed between each cluster and all all other clusters in the training set (“LR” method, animal sex as latent variable), and the union of

significantly differential genes (average $log_2FC > 0.5$ and Bonferonni-adjusted p-value < 0.001) was used as the feature set for model fitting. Other training parameters, including usage of an offset in each model to adjust for observed frequencies of cell types, strategy for selection of lambda, an alpha value of 0.99, and 10-fold cross validation, were kept as previously described (1).

We next used these models to calculate predicted similarity of tumor clusters (split by sample), tumor-associated fibroblasts, and tumor-associated endothelial cells to each normal kidney cell type as previously described (1). The predicted logits were averaged within each cluster in the test set, converted to probabilities, and visualized in a heatmap. Rows representing normal kidney clusters with low cell numbers, wherein during model training convergence for a 2nd lamba value was not reached after 100,000 iterations, were omitted from the visualization.

Determination of PT2399, anti-PD-1 and anti-CTLA4 in animal tissues

Determination of murine anti-PD-1 (muDX400) & anti-CTLA4 in mouse serum: Levels of therapeutic antibodies were evaluated by a bioanalytical method using the MSD electrochemiluminescence (ECL) format. The minimal detectable concentration of muDX400 (murine anti-PD-1) and anti-CTLA4 in serum was 10 ng/mL and 19.5 ng/ml respectively. Briefly, MSD highbind 96-well plates were coated with recombinant mouse PD1/Fc chimera or mouse CTLA4. Plates were blocked with 5% BSA prior to incubation of standards, controls and samples, with washes in between each step. ECL was measured after incubation of detection reagent on the MSD Sector Imager S600. The resulting ECL signal produced was proportional to the concentration of muDX400 or anti-CTLA4 in the samples, quantitatively measured by comparison with a standard curve.

Determination of PT2399 in mouse blood and tumor: Mouse biological samples (blood and tumor homogenate) were prepared with protein precipitation using Hamilton Microlab STAR Automated Liquid Handling System. In brief, 50 µl of the bio-sample was mixed with 200 µl acetonitrile (containing the internal standard), which was vortexed for 10 minutes before being centrifuged at 4,000xg for 10 minutes at 4°C. The upper layer was transferred to a new plate for LC-MS/MS injection, and the bottom precipitation was discarded. Samples were analyzed with LC-MS/MS (Waters ACQUITY UPLC System and a Sciex 6500 Triple-Quad mass spectrometer) run in negative electrospray ionization (ESI-) mode. The minimal detectable concentration of PT2399 in mouse samples was 1nM.

Immunohistochemistry of tumor tissues

Immunohistochemical staining of tumors to assess gene editing was performed as follows:
PBRM1: IHC was performed on the LeicaBiosystems' BondRX autostainer with the following conditions: Epitope Retrieval 2 (EDTA) 30', PBRM1 (Bethyl #A301-591A, 1:5000 60'), and the Bond Polymer Refine Detection Kit (LeicaBiosystems #DS9800) with omission of the PostPrimary reagent. Isotype control reagent was used in place of primary antibody for the negative control. Slides were removed from the Bond autostainer, dehydrated through ethanols, cleared with xylenes, and coverslipped.

Manual staining was performed for pS6, GFP, PAX8, and CA9 as follows:
S6 Ribosomal Protein (phospho): antigen retrieval with citrate buffer (Vector Labs, 10' at 100C), pS6 (Cell Signaling Technology #4857, 1:150 overnight @ 4C), biotinylated secondary antibody goat anti-rabbit IgG (Vector Labs), ABC Elite (Vector Labs), and DAB.
GFP: antigen retrieval with proteinase K (Agilent #S3020, 5' RT), GFP (abcam #ab6556, 1:4000 overnight @ 4C), biotinylated secondary antibody goat anti-rabbit IgG (Vector Labs), ABC Elite (Vector Labs), and DAB.
PAX8: antigen retrieval with EDTA pH8.0 (Epredia™, 30" at 125C), PAX8 (ProteinTech Group #10336-1-AP, 1:1000 60'), ImmPRESS® HRP anti-Rabbit IgG Polymer Kit (Vector Labs), and DAB.
Carbonic Anhydrase IX (CA9): antigen retrieval with citrate buffer (Vector Labs, 30" at 125C), CA9 (R&D Systems #AF2344, 1:100 60'), Rabbit anti-Goat IgG (Vector Labs), ImmPRESS® HRP anti-Rabbit IgG Polymer Kit (Vector Labs), and DAB.
Sections were counterstained with hematoxylin, dehydrated through ethanols, cleared with xylenes, and coverslipped. H&E slides were stained on the Tissue-Tek® Prisma™ autostainer.

Immunohistochemical staining of tumors from the multi-drug efficacy study was performed on formalin-fixed paraffin-embedded tissue slides in a Leica Biosystems BondRX autostainer with the following conditions: Epitope Retrieval 1 (Citrate) 20 min for Cyclin D1, Ki67, CD8a, CD3 sections, and Epitope Retrieval 2 (EDTA) 10 min for CD31 sections. Antibody source and conditions were as follows: Cyclin D1 (Cell Signaling Technology #2978, 1:200 incubated 30 min), Ki67 (Cell Signaling Technology #12202, 1:200 incubated 30 min), CD8a (eBioscience #14-0195-82, 1:50 incubated 30 min), CD3 (Bio-Rad #MCA1477, 1:100 incubated 60 min), CD31 (abcam #ab28364, 1:100 incubated 60 min), and the Bond Polymer Refine Detection Kit (LeicaBiosystems #DS9800). For CD8a and CD3 a secondary antibody Rabbit anti-Rat IgG (Vector Laboratories) was also used. Isotype control reagents were used in place of primary antibodies for the negative controls. Slides were removed from the Bond autostainer, dehydrated through ethanol series, cleared with xylenes, and coverslipped. Images were captured using the Leica Aperio At2 scanner. Image analysis for Ki67, CyclinD1, CD31, CD8a and CD3 was accomplished using CytoNuclear algorithm in Halo imaging analysis software (v3.5.3577.300; Indica Labs, Albuquerque, NM). CD31 positive vessels were quantified using Aperio vasculature algorithm.

Image annotations were performed by a pathologist (A.J). Fields were excluded if they contained areas of artifact such as folds or tears or significant necrosis.

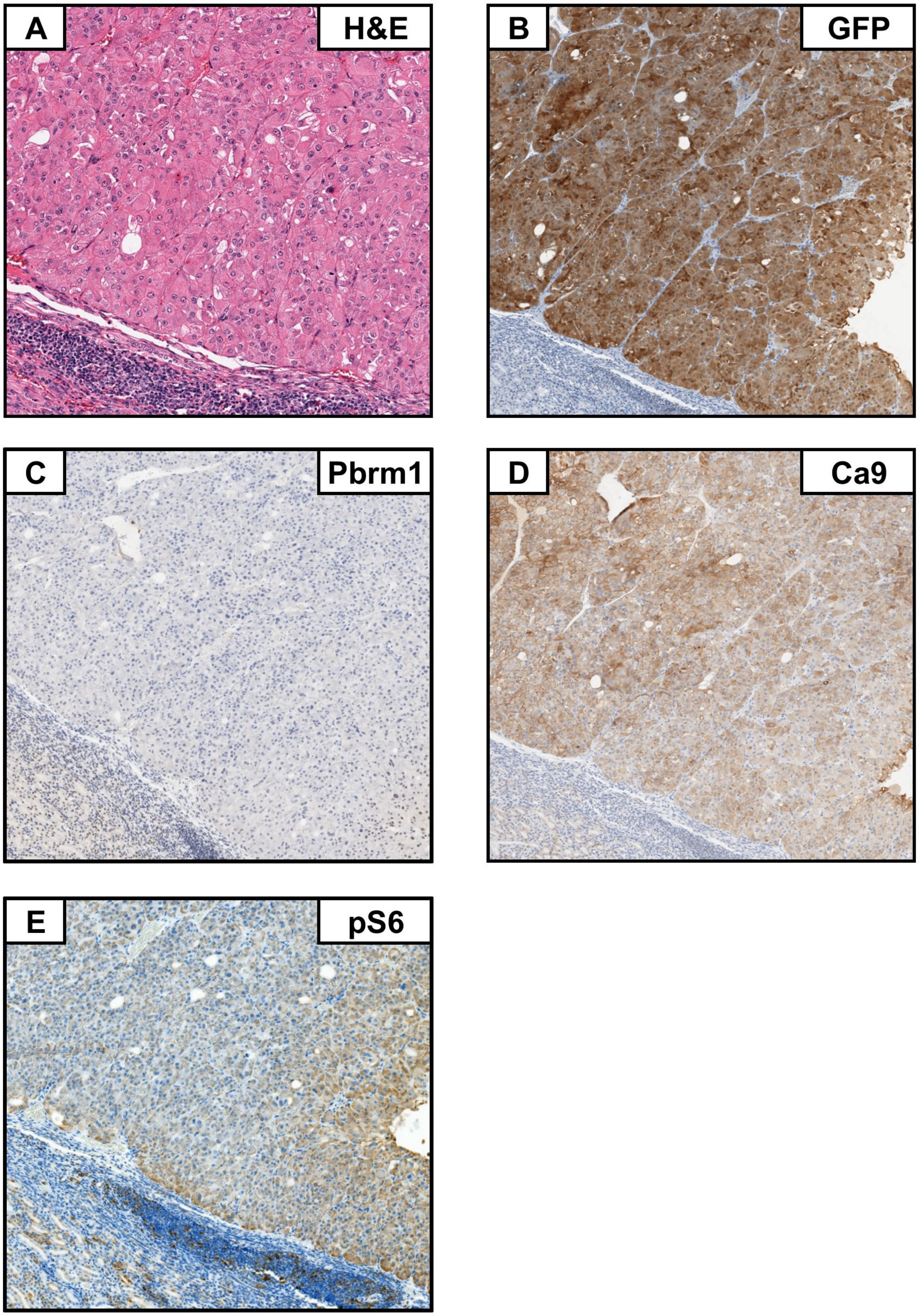

Figure S1. Immunohistochemistry of biomarkers indicating targeted gene editing.
A) H&E staining of Ksp-Cre AAV model kidney tumor with adjacent normal kidney (lower left region) showing a cystic-solid histology. Immunohistochemical staining of Ksp-Cre AAV model for B) GFP, C) Baf180 (Pbrm1), D) Ca9, and E) phospho-S6 (mTOR readout).

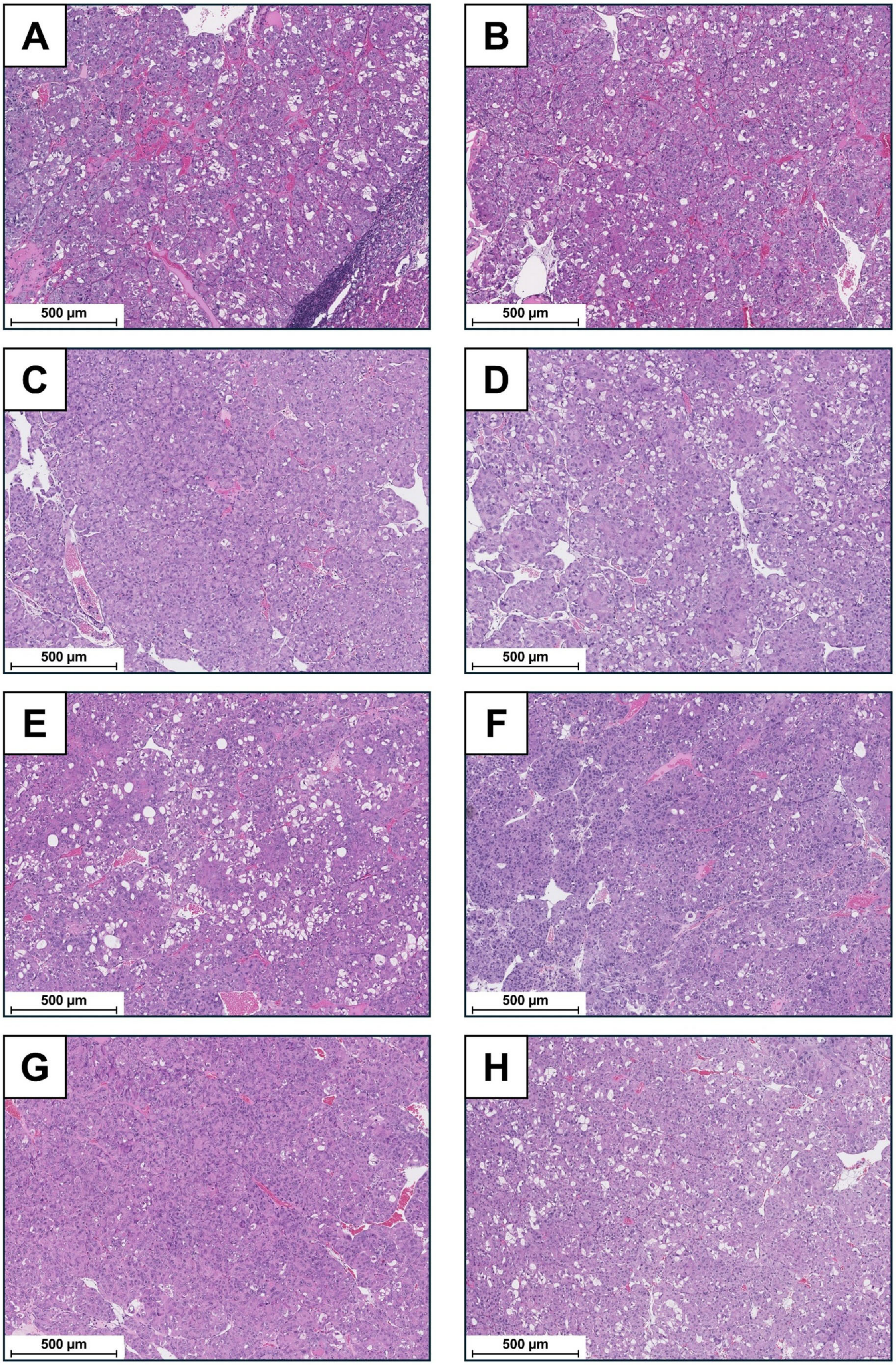
A
500 µm
B
500 µm
C
500 µm
D
500 µm
E
500 µm
F
500 µm
G
500 µm
H
500 µm

Figure S2. Examples of kidney tumor histology in the Cre-less AAV model.

Two different kidney tumor regions were selected from each of four different Cre-less AAV model mice to demonstrate the variable histology. H&E staining is shown. A,B) mouse 1; C,D) mouse 2; E,F) mouse 3; G,H) mouse 4. Scale bar, 500 µm.

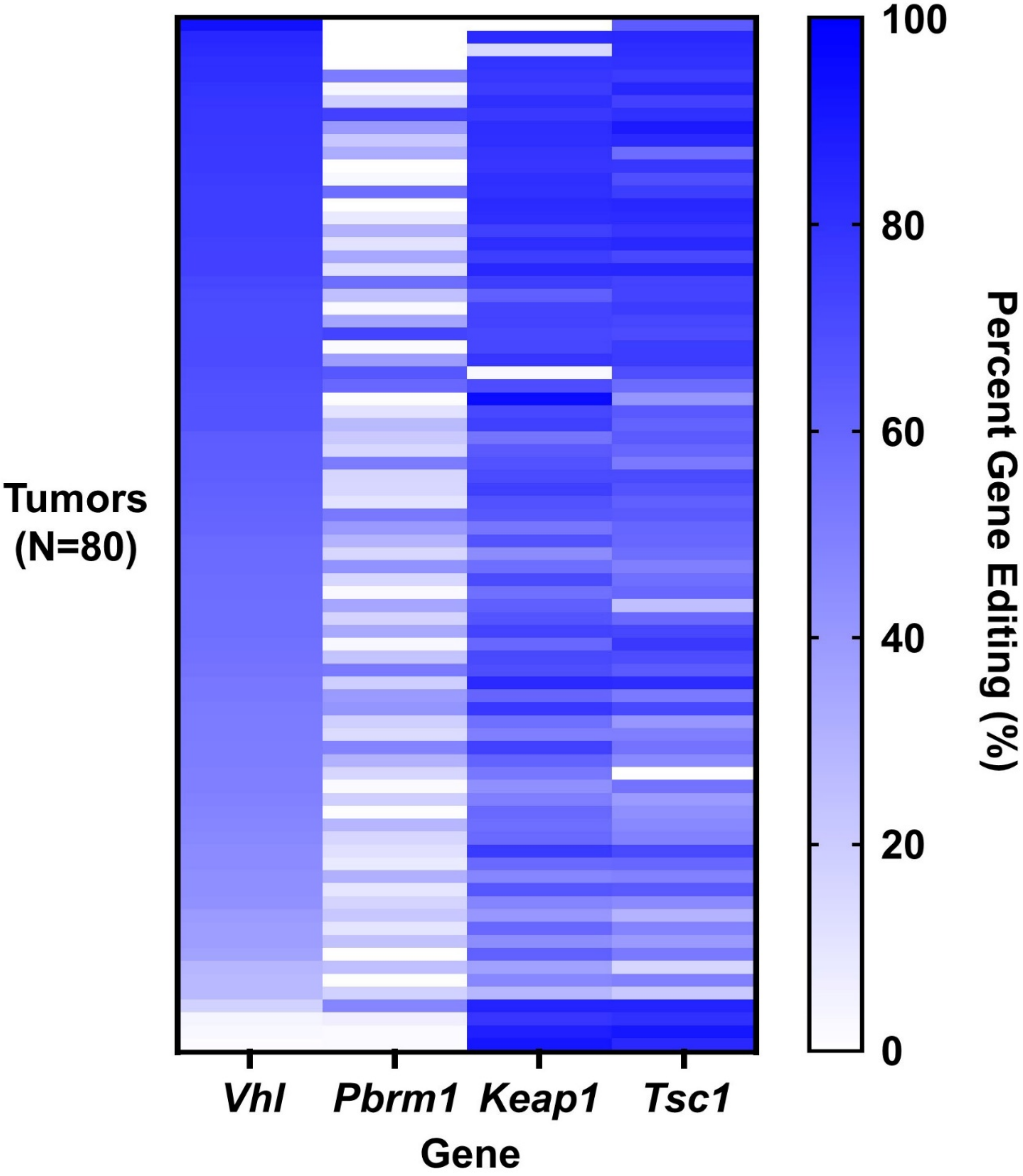

Figure S3. Percent CRISPR-Cas9 gene editing of multi-drug study tumors for *Vhl, Pbrm1, Keap1,* and *Tsc1*.

Percent CRISPR-Cas9 gene editing of *Vhl, Pbrm1, Keap1* and *Tsc1* was performed by PCR-based sequencing in 80 multi-drug study kidney tumors from Cre-less AAV model.

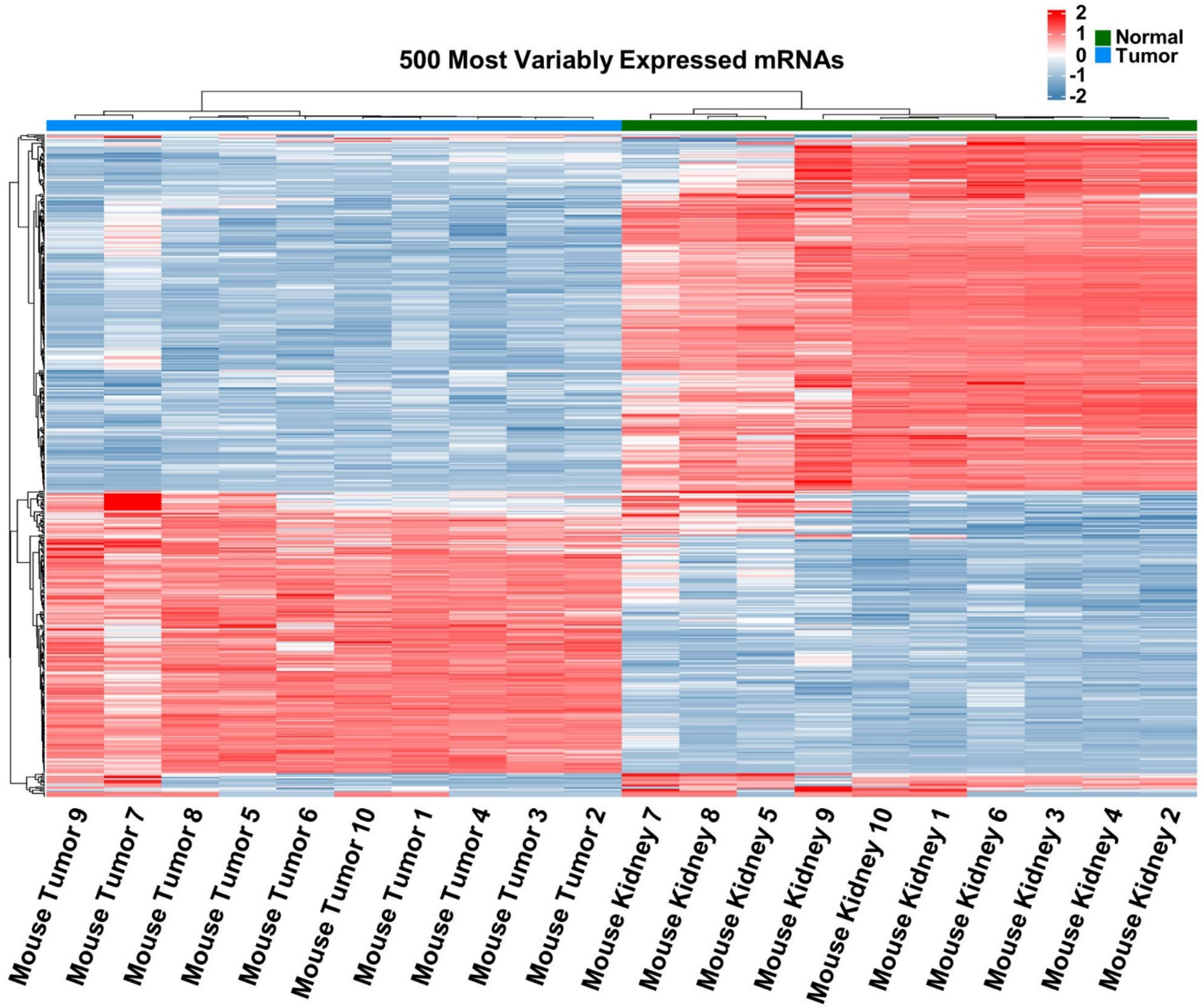

Figure S4. Heatmap of 500 most variable genes in the Cre-less AAV model kidney tumors vs. normal mouse kidney.

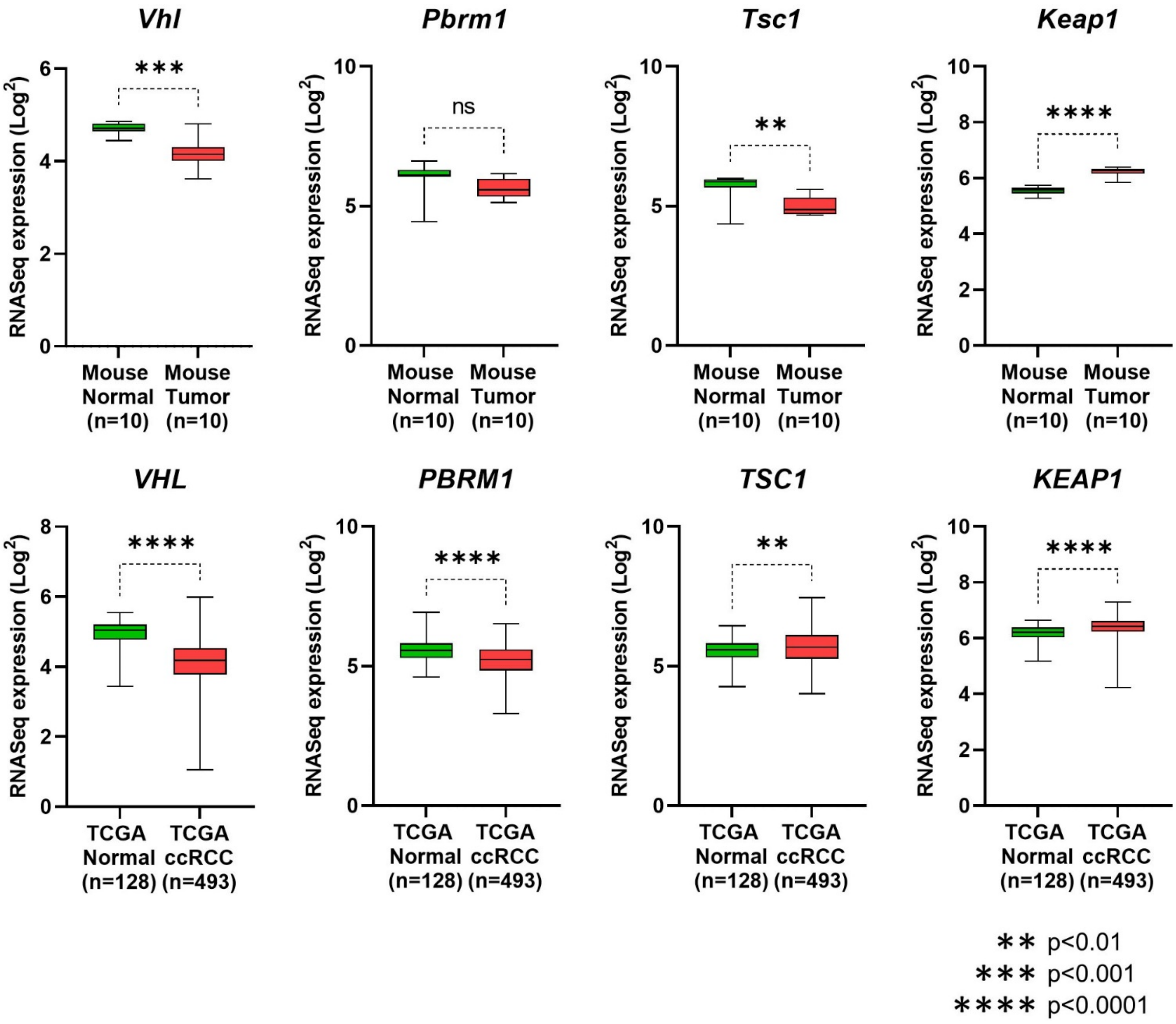

Figure S5. Expression analysis of gene edited targets.

Upper panels: Box plots of the RNAseq-based expression data for *Vhl*, *Pbrm1*, *Tsc1*, and *Keap1* comparing 10 mouse tumors to 10 normal mouse kidney samples. Data presented as log^2 expression values and compared by Welch's t test.

Lower panels: Box plots of the TCGA expression data for *VHL*, *PBRM1*, *TSC1*, and *KEAP1* comparing 493 human ccRCC tumors to 128 normal human kidney samples. Data presented as log^2 expression values and compared by Welch's t test.

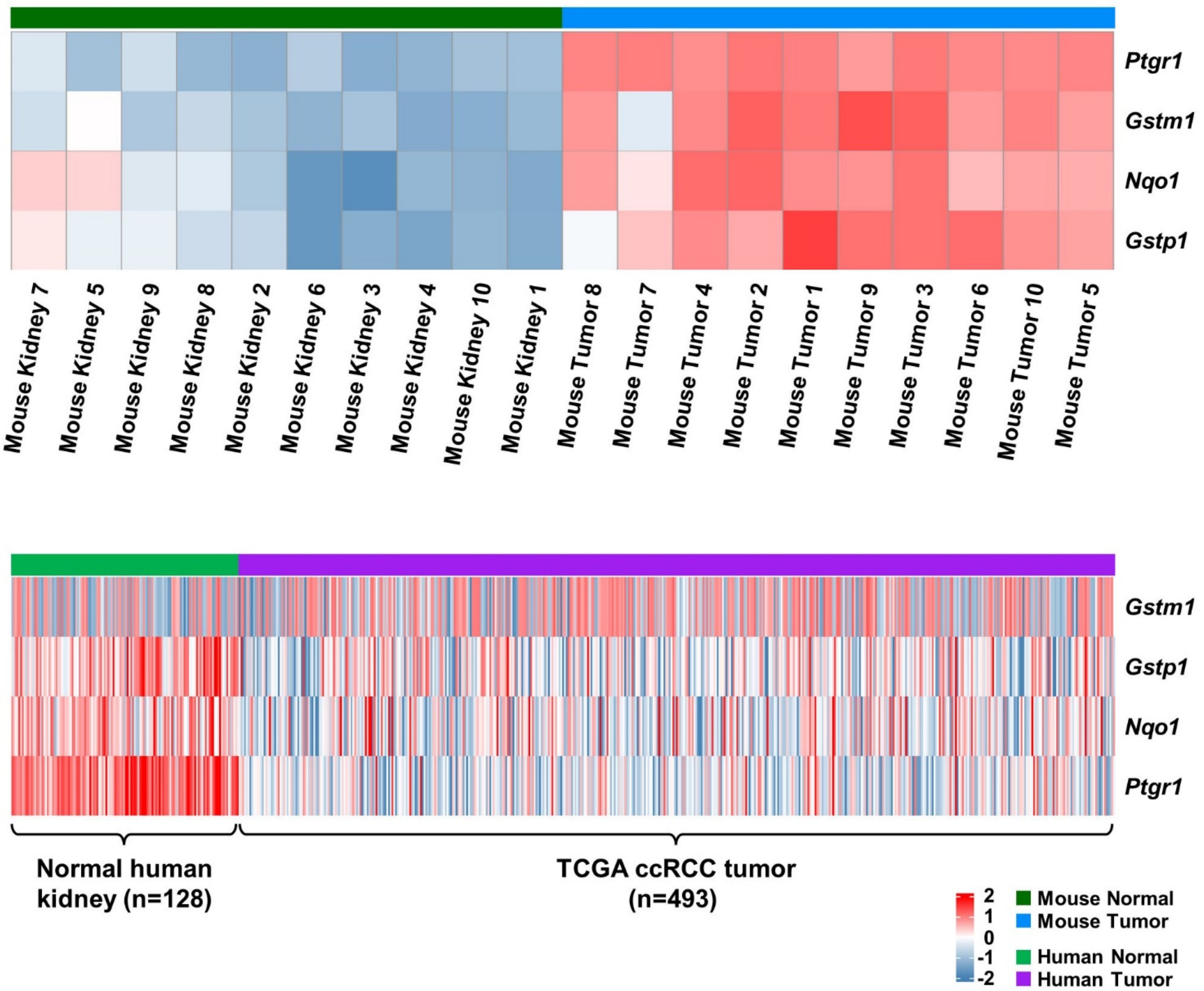

Figure S6. Analysis of Nrf2 target genes predicted to be upregulated by *Keap1* loss.
Upper panel: heatmap comparing mouse normal kidney and mouse tumor from Cre-less AAV model. Note: mouse gene sets do not have a specific Nrf2 pathway gene set, so representative Nrf2 target genes were selected. Lower panel: heatmap comparing ccRCC from the TCGA KIRC dataset with normal human kidney. TCGA ccRCC tumors show little evidence of increased expression of the NRF2 pathway genes in comparison to normal kidney.

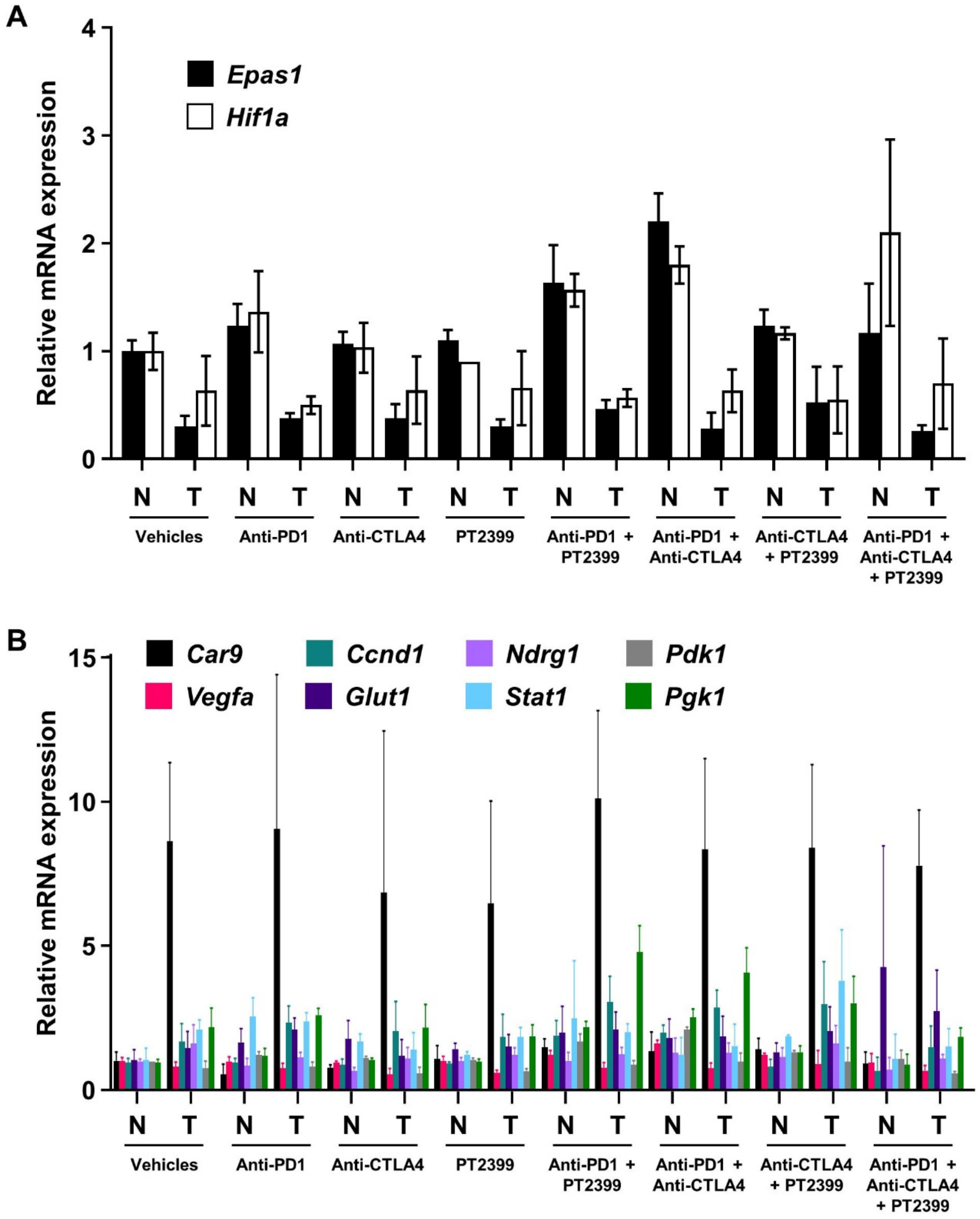

Figure S7. Gene expression of HIF targets in kidney tumors from efficacy study of PT2399 and immune checkpoint inhibitors.

A) *HIF-1a* and *Epas1*(HIF-2a) expression in normal kidney (N) and tumor tissue (T) from Cre-less AAV model mice as determined by qPCR. N=5 mice for each treatment arm collected after 4 days of treatment. B) HIF target gene mRNA expression in normal kidney (N) and tumor (T) tissue from Cre-less AAV model mice as determined by qPCR. N=5 mice for each treatment arm collected after 4 days of treatment.

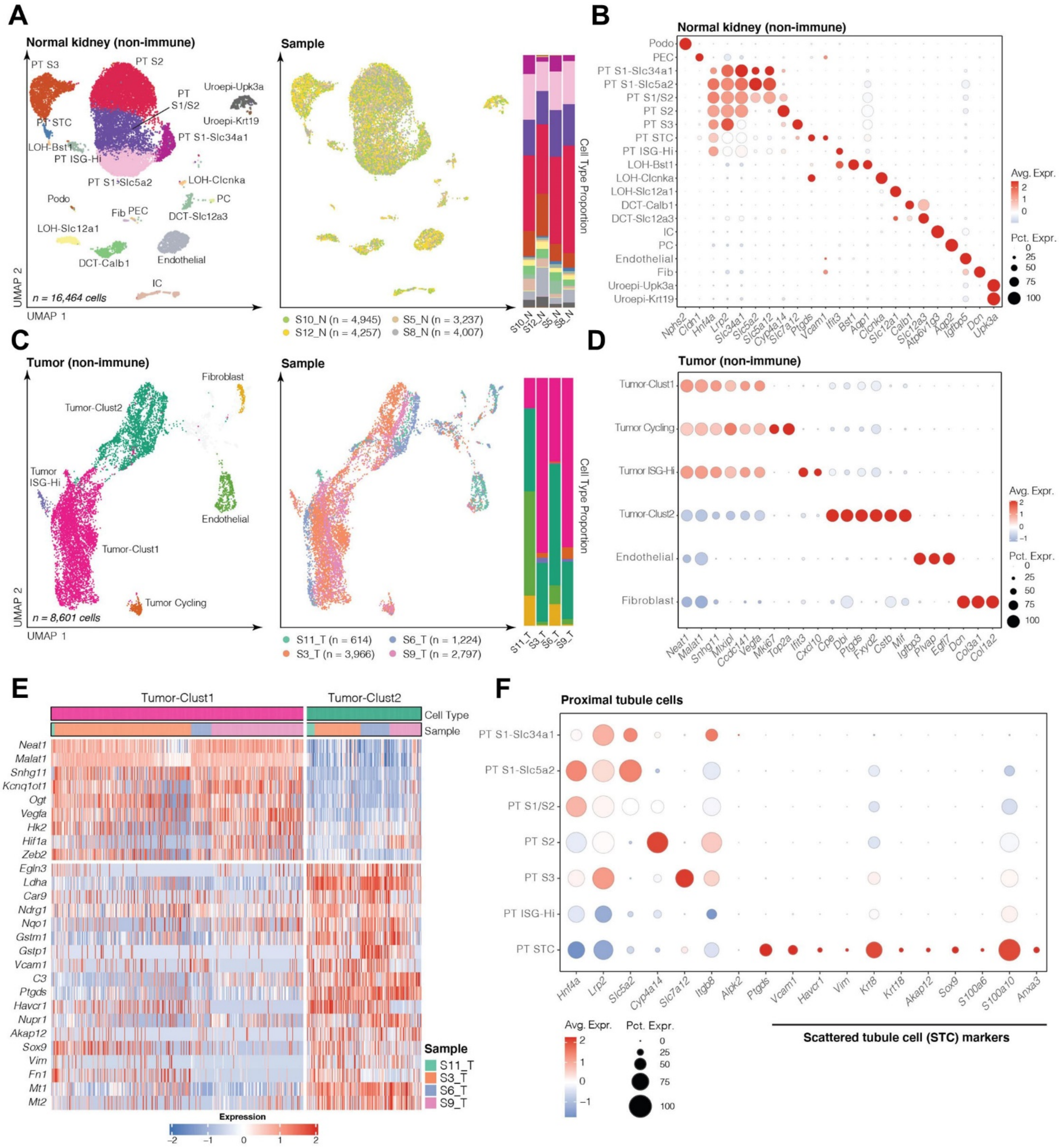
A
Normal kidney (non-immune)
PT S3
PT S2
PT S1/S2
Uroepi-Upk3a
Uroepi-Krt19
PT STC
PT S1-Slc34a1
LOH-Bst1
PT ISG-Hi
PT S1-Slc5a2
LOH-Clcnka
PC
Podo
Fib
PEC
DCT-Slc12a3
LOH-Slc12a1
DCT-Calb1
Endothelial
IC
n = 16,464 cells
UMAP 2
UMAP 1
Sample
S10_N (n = 4,945)
S12_N (n = 4,257)
S5_N (n = 3,237)
S8_N (n = 4,007)
S10_N
S12_N
S5_N
S8_N
Cell Type Proportion
B
Normal kidney (non-immune)
Podo
PEC
PT S1-Slc34a1
PT S1-Slc5a2
PT S1/S2
PT S2
PT S3
PT STC
PT ISG-Hi
LOH-Bst1
LOH-Clcnka
LOH-Slc12a1
DCT-Calb1
DCT-Slc12a3
IC
PC
Endothelial
Fib
Uroepi-Upk3a
Uroepi-Krt19
Nphs2
Cldn1
Hnf4a
Lrp2
Slc34a1
Slc5a2
Slc5a12
Cyp4a14
Slc7a12
Ptgds
Vcam1
Ifit3
Bst1
Aqp1
Clcnka
Slc12a1
Calb1
Slc12a3
Atp6v1g3
Aqp2
Igfbp5
Dcn
Upk3a
Avg. Expr.
2
1
0
Pct. Expr.
0
25
50
75
100
C
Tumor (non-immune)
Fibroblast
Tumor-Clust2
Tumor ISG-Hi
Endothelial
Tumor-Clust1
Tumor Cycling
n = 8,601 cells
UMAP 2
UMAP 1
Sample
S11_T (n = 614)
S3_T (n = 3,966)
S6_T (n = 1,224)
S9_T (n = 2,797)
S11_T
S3_T
S6_T
S9_T
Cell Type Proportion
D
Tumor (non-immune)
Tumor-Clust1
Tumor Cycling
Tumor ISG-Hi
Tumor-Clust2
Endothelial
Fibroblast
Neat1
Malat1
Snhg11
Mlxipl
Ccdc141
Vegfa
Mki67
Top2a
Ifit3
Cxcl10
Cpe
Dbi
Ptgds
Fxyd2
Cstb
Mif
Igfbp3
Plvap
Egfl7
Dcn
Col3a1
Col1a2
Avg. Expr.
2
1
0
−1
Pct. Expr.
0
25
50
75
100
E
Tumor-Clust1
Tumor-Clust2
Cell Type
Sample
Neat1
Malat1
Snhg11
Kcnq1ot1
Ogt
Vegfa
Hk2
Hif1a
Zeb2
Egln3
Ldha
Car9
Ndrg1
Nqo1
Gstm1
Gstp1
Vcam1
C3
Ptgds
Havcr1
Nupr1
Akap12
Sox9
Vim
Fn1
Mt1
Mt2
Sample
S11_T
S3_T
S6_T
S9_T
Expression
−2
−1
0
1
2
F
Proximal tubule cells
PT S1-Slc34a1
PT S1-Slc5a2
PT S1/S2
PT S2
PT S3
PT ISG-Hi
PT STC
Hnf4a
Lrp2
Slc5a2
Cyp4a14
Slc7a12
Itgb8
Alpk2
Ptgds
Vcam1
Havcr1
Vim
Krt8
Krt18
Akap12
Sox9
S100a6
S100a10
Anxa3
Scattered tubule cell (STC) markers
Avg. Expr.
2
1
0
−1
Pct. Expr.
0
25
50
75
100

Figure S8. Key phenotypic markers and cluster-specific genes in normal mouse kidney and Cre-less AAV tumor scRNA-Seq data.

A) UMAP of non-immune cells from 4 normal kidney samples, colored and labelled by cluster/cell type (left) and sample (middle). Stacked bar plots (right) show cell type proportional makeup of each sample. B) Dotplot showing expression of key phenotypic markers and cluster-specific genes in normal kidney cell types. Dot size represents percentage of cells in cluster with non-zero expression of a given gene. Dot color represents average expression of a gene within a cluster. C) UMAP of non-immune cells from 4 Cre-less AAV kidney tumors, colored and labelled by cluster/cell type (left) and sample (middle). Stacked bar plots (right) show cell type proportional makeup of each sample. D) Dotplot showing expression of key phenotypic markers and cluster-specific genes in tumor clusters and tumor-associated stromal cell types. E) Heatmap of selected genes with significant differential expression between Tumor-Clust1 and Tumor-Clust2 cells. F) Dotplot showing expression of pan-PT, PT S1, PT S2, PT S3, and scattered tubule cell markers in proximal tubule cells only.

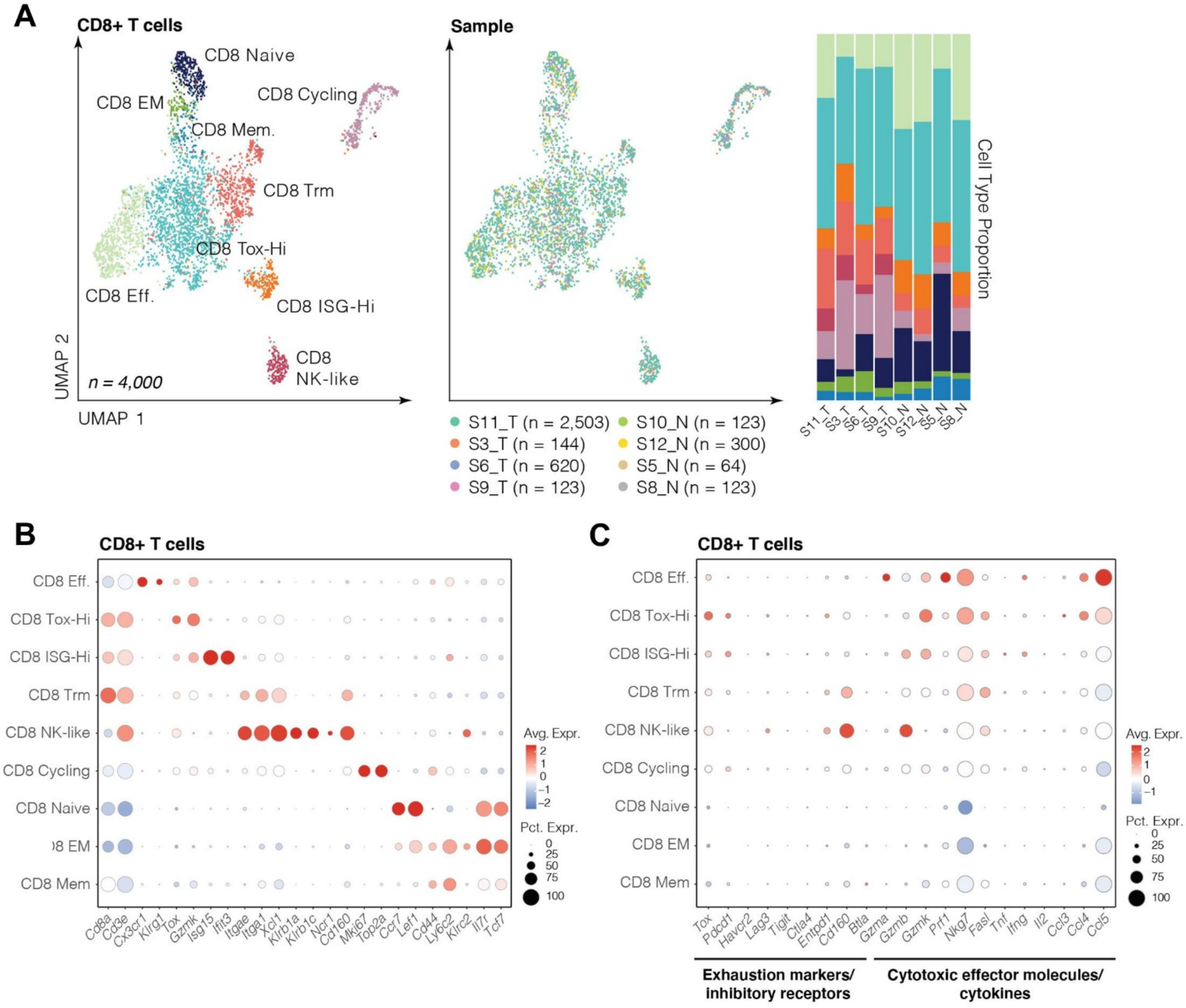

Figure S9. Key phenotypic markers and cluster-specific genes in CD8+ T cells from normal mouse kidney and Cre-less AAV tumors.

A) UMAP of CD8+ T cells from 4 normal kidney and 4 tumor samples, colored and labelled by cluster/cell type (left) and sample (middle). Stacked bar plots (right) show cell type proportional makeup of each sample. B) Dotplot showing expression of key phenotypic markers and cluster-specific genes in CD8+ T cells. C) Dotplot showing expression of exhaustion markers/inhibitory receptors and cytotoxic effector molecules/cytokines in CD8+ T cells.

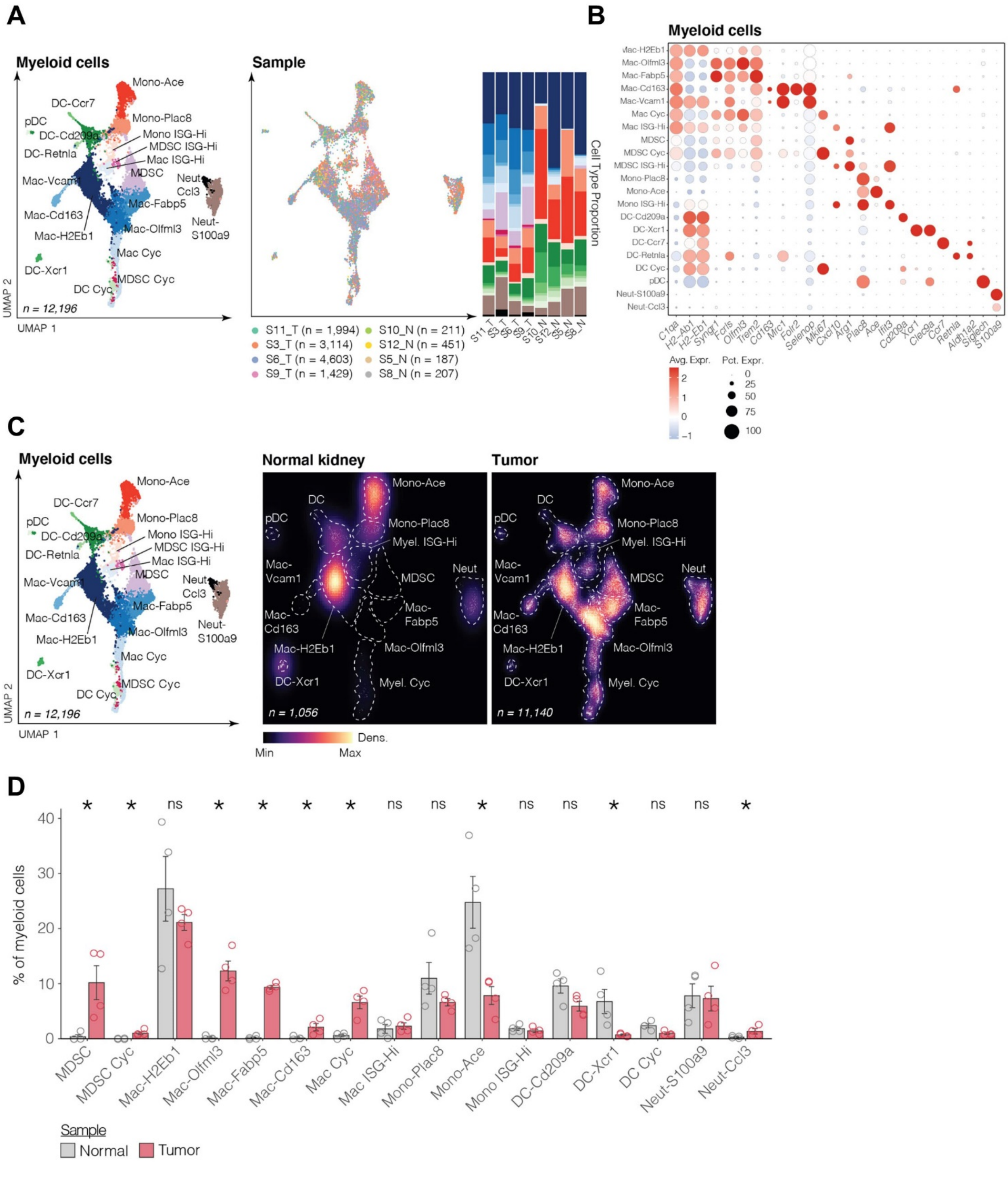
A
Myeloid cells
Mono-Ace
DC-Ccr7
Mono-Plac8
pDC
DC-Cd209a
Mono ISG-Hi
MDSC ISG-Hi
DC-Retnla
Mac ISG-Hi
MDSC
Mac-Vcam1
Neut-Ccl3
Mac-Fabp5
Mac-Cd163
Mac-Olfml3
Mac-H2Eb1
Neut-S100a9
Mac Cyc
DC-Xcr1
MDSC Cyc
DC Cyc
n = 12,196
UMAP 1
UMAP 2
Sample
S11_T (n = 1,994)
S3_T (n = 3,114)
S6_T (n = 4,603)
S9_T (n = 1,429)
S10_N (n = 211)
S12_N (n = 451)
S5_N (n = 187)
S8_N (n = 207)
Cell Type Proportion
B
Myeloid cells
Avg. Expr.
Pct. Expr.
C
Myeloid cells
Normal kidney
Tumor
Myel. ISG-Hi
Myel. Cyc
Neut
n = 1,056
n = 11,140
Dens.
Min
Max
D
% of myeloid cells
Sample
Normal
Tumor

Figure S10. Key phenotypic markers and cluster-specific genes in myeloid cells from normal mouse kidney and Cre-less AAV tumors.

A) UMAP of myeloid cells from 4 normal kidney and 4 tumor samples, colored and labelled by cluster/cell type (left) and sample (middle). Stacked bar plots (right) show cell type proportional makeup of each sample. B) Dotplot showing expression of key phenotypic markers and cluster-specific genes in myeloid cells. C) Left, UMAP of myeloid cells from all 4 normal kidney and 4 tumor samples, colored and labelled by cluster/cell type. Middle and right, UMAPs of myeloid cells split by normal kidney and tumor samples and colored by relative point density. D) Quantification of myeloid subset proportions relative to total myeloid cells in normal kidney and tumor samples. Only subsets with at least 100 total cells are shown. P-values determined by two-sided Wilcoxon rank-sum test.

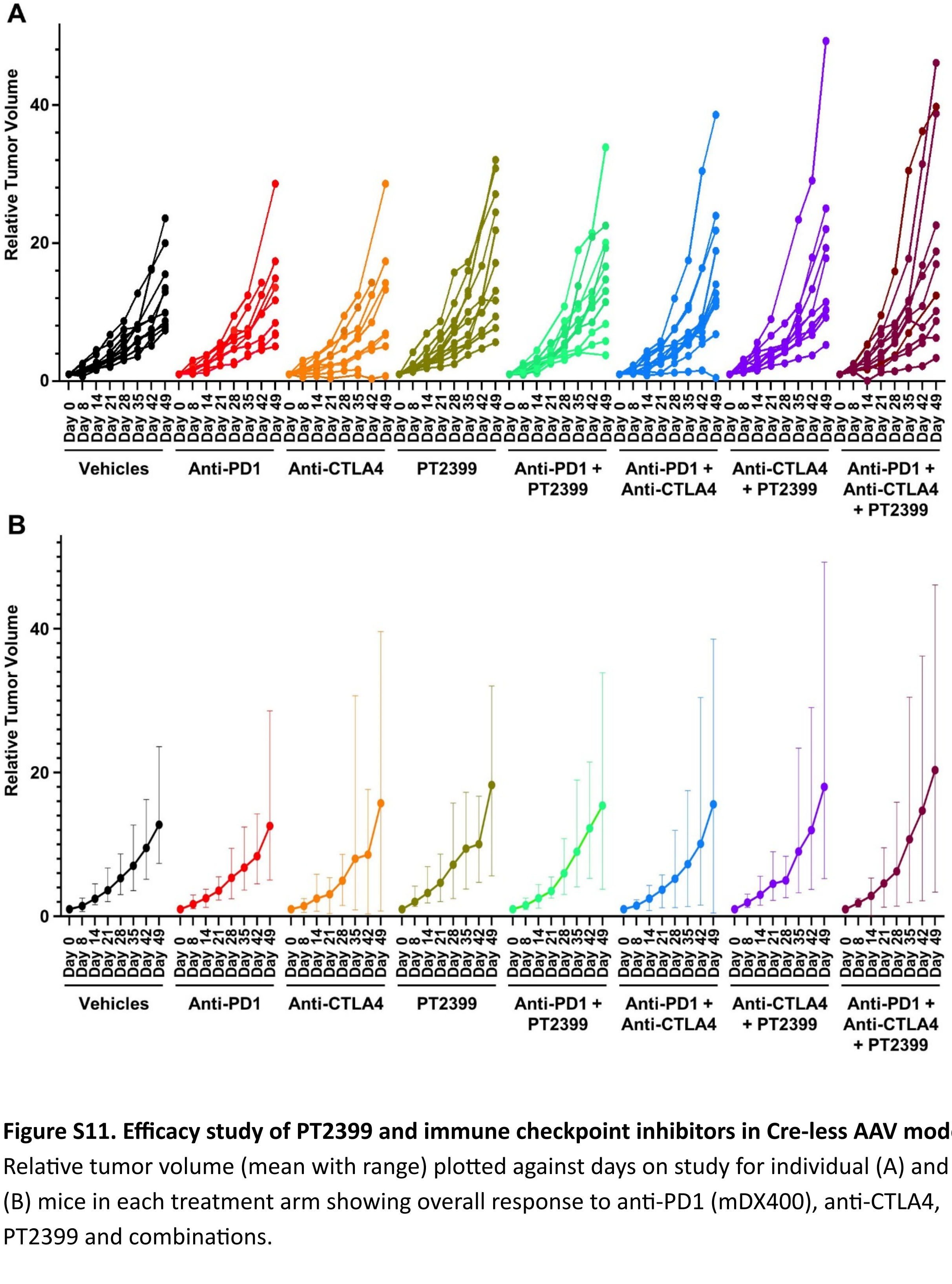

Figure S11. Efficacy study of PT2399 and immune checkpoint inhibitors in Cre-less AAV model. Relative tumor volume (mean with range) plotted against days on study for individual (A) and all (B) mice in each treatment arm showing overall response to anti-PD1 (mDX400), anti-CTLA4, PT2399 and combinations.

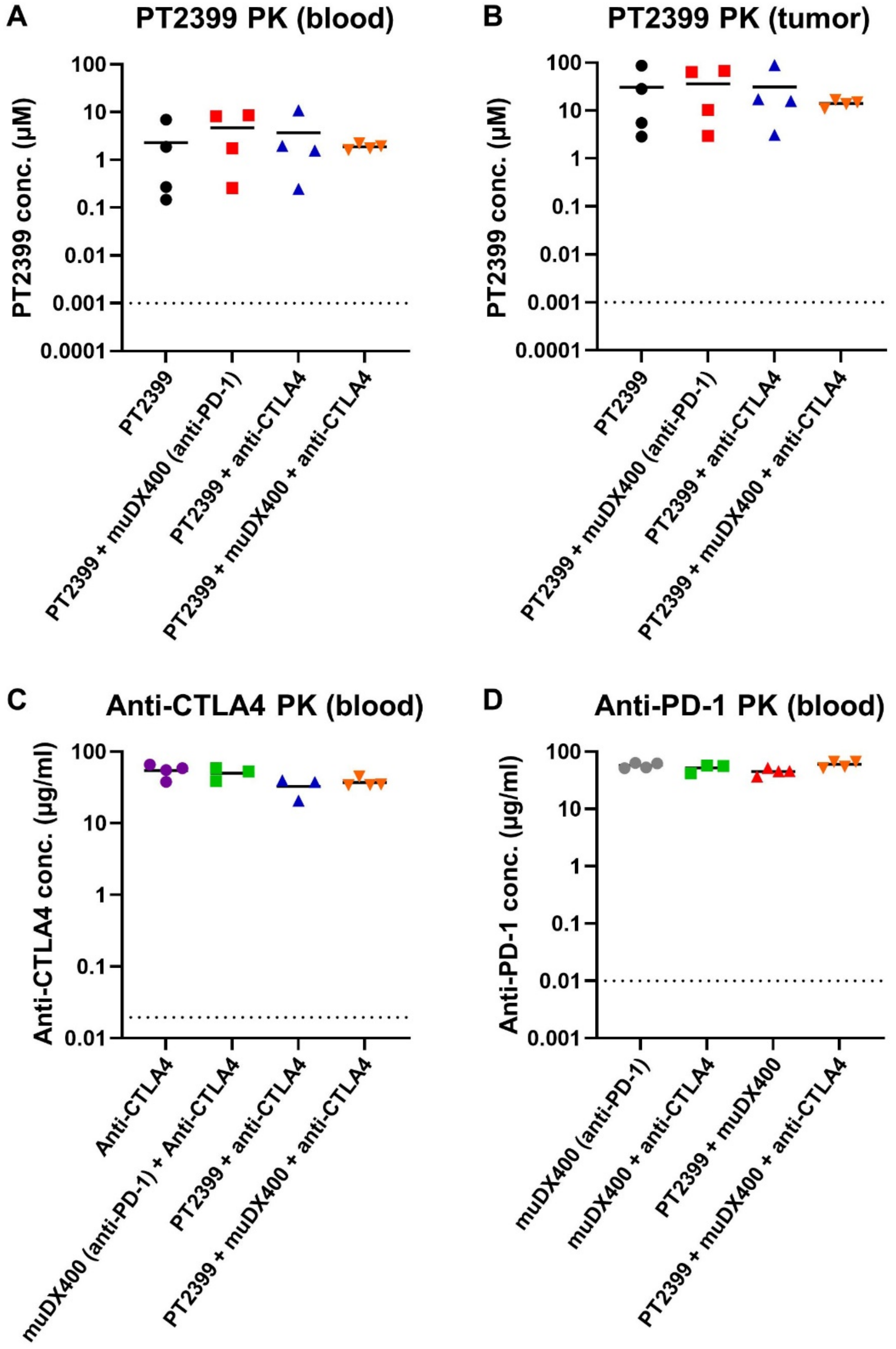

Figure S12. Determination of PT2399, anti-PD1 and anti-CTLA4 levels in blood and tumor from Cre-less AAV model treated with immunotherapy and PT2399.

PT2399 levels in blood (A) and tumors (B) from mice in treatment arms as indicated. C) anti-CTLA4 in blood from mice in treatment arms as indicated. D) anti-PD1 in blood from mice in treatment arms as indicated. N=5 mice per treatment arm, collected after 4 days of treatment.

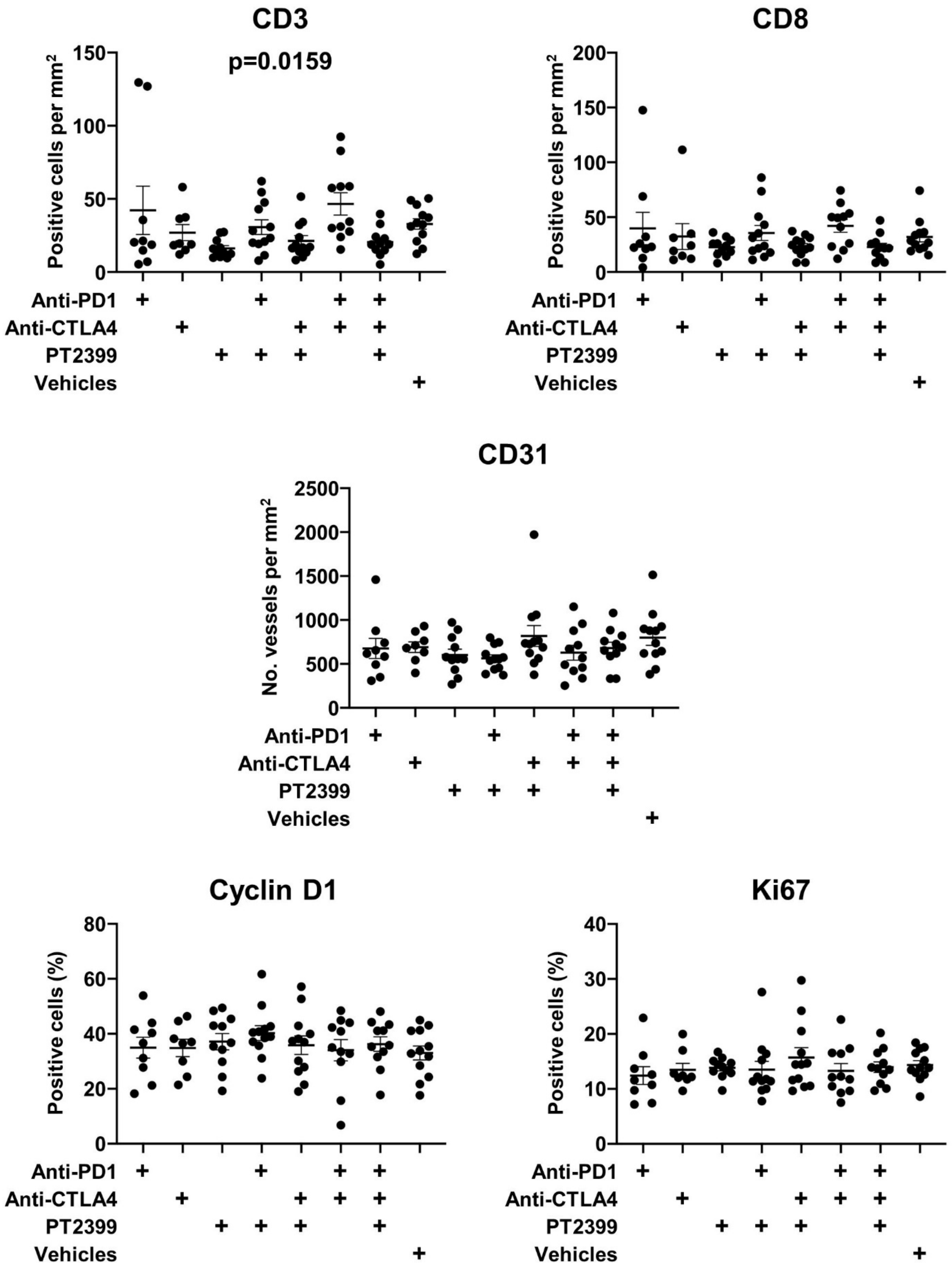
CD3
p=0.0159
Positive cells per mm2
150
100
50
0
CD8
Positive cells per mm2
200
150
100
50
0
CD31
No. vessels per mm2
2500
2000
1500
1000
500
0
Cyclin D1
Positive cells (%)
80
60
40
20
0
Ki67
Positive cells (%)
40
30
20
10
0
Anti-PD1
Anti-CTLA4
PT2399
Vehicles

Figure S13. Immunohistochemical analysis of biomarkers in kidney tumors from efficacy study of PT2399 and checkpoint inhibitors in Cre-less AAV model.

CD3, CD8, CD31, cyclin D and Ki67 were evaluated by immunohistochemistry in 10 mice from each treatment arm. P values were determined by ordinary one-way ANOVA. p-value for CD3, 0.0159; all others, ns (non-significant).

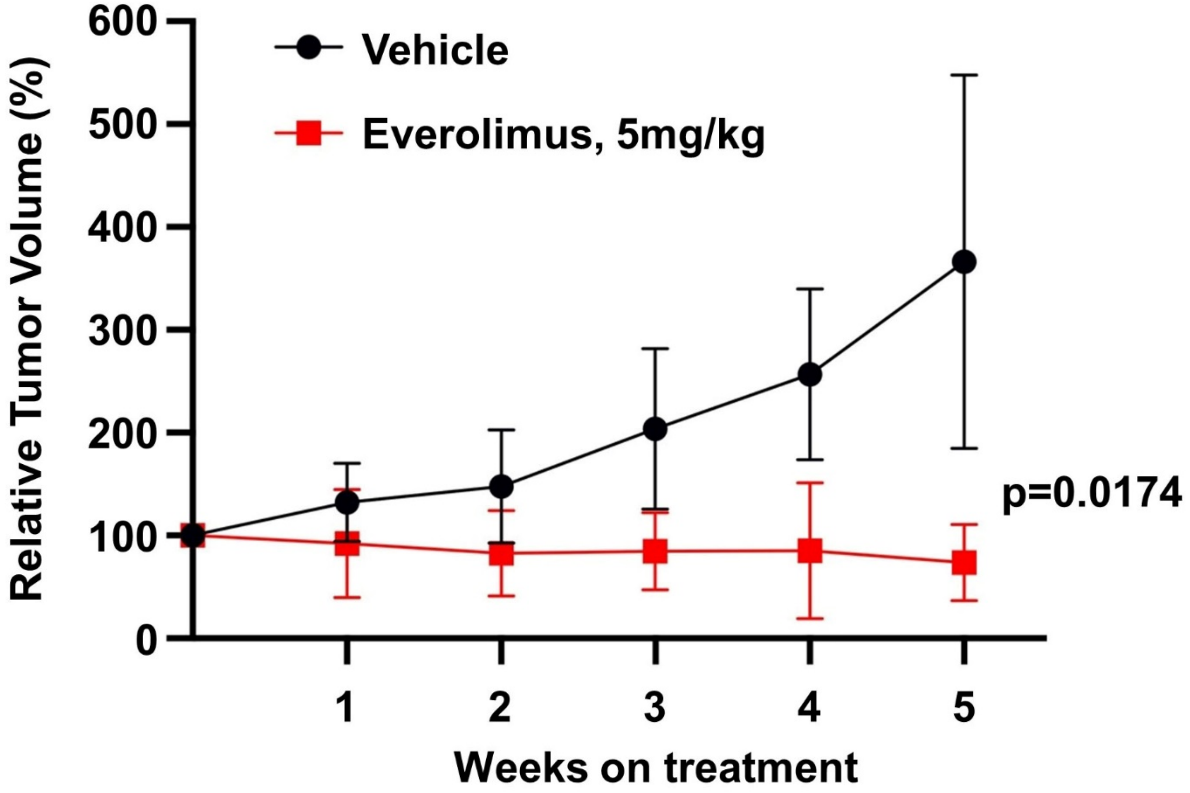

Figure S14. Tumor response to mTOR inhibition in Cre-less AAV model.
Everolimus treatment (5mg/kg, p.o., 5 x week) resulted in robust inhibition of tumor growth compared to vehicle (74% vs 366% relative tumor volume after 5 weeks on treatment; p=0.0174).

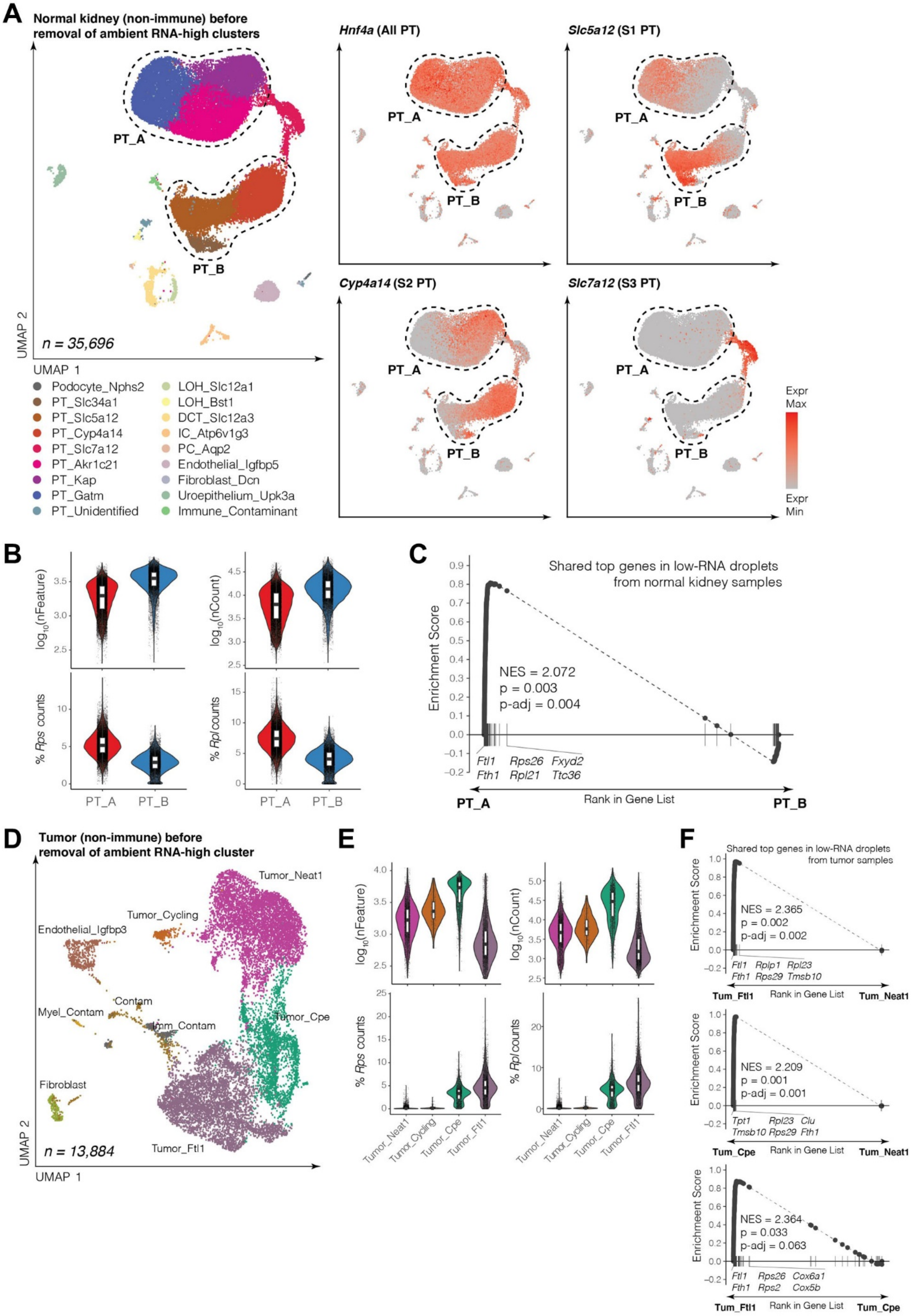
A
Normal kidney (non-immune) before removal of ambient RNA-high clusters
PT_A
PT_B
n = 35,696
UMAP 1
UMAP 2
Podocyte_Nphs2
PT_Slc34a1
PT_Slc5a12
PT_Cyp4a14
PT_Slc7a12
PT_Akr1c21
PT_Kap
PT_Gatm
PT_Unidentified
LOH_Slc12a1
LOH_Bst1
DCT_Slc12a3
IC_Atp6v1g3
PC_Aqp2
Endothelial_Igfbp5
Fibroblast_Dcn
Uroepithelium_Upk3a
Immune_Contaminant
Hnf4a (All PT)
Slc5a12 (S1 PT)
Cyp4a14 (S2 PT)
Slc7a12 (S3 PT)
Expr Max
Expr Min
B
log10(nFeature)
log10(nCount)
% Rps counts
% Rpl counts
PT_A
PT_B
C
Shared top genes in low-RNA droplets from normal kidney samples
Enrichment Score
NES = 2.072
p = 0.003
p-adj = 0.004
Ftl1
Fth1
Rps26
Rpl21
Fxyd2
Ttc36
PT_A
Rank in Gene List
PT_B
D
Tumor (non-immune) before removal of ambient RNA-high cluster
Tumor_Neat1
Tumor_Cycling
Endothelial_Igfbp3
Contam
Myel_Contam
Imm_Contam
Tumor_Cpe
Fibroblast
Tumor_Ftl1
n = 13,884
UMAP 1
UMAP 2
E
log10(nFeature)
log10(nCount)
% Rps counts
% Rpl counts
Tumor_Neat1
Tumor_Cycling
Tumor_Cpe
Tumor_Ftl1
F
Shared top genes in low-RNA droplets from tumor samples
Enrichment Score
NES = 2.365
p = 0.002
p-adj = 0.002
Ftl1
Fth1
Rplp1
Rps29
Rpl23
Tmsb10
Tum_Ftl1
Rank in Gene List
Tum_Neat1
NES = 2.209
p = 0.001
p-adj = 0.001
Tpt1
Tmsb10
Rpl23
Rps29
Clu
Fth1
Tum_Cpe
Rank in Gene List
Tum_Neat1
NES = 2.364
p = 0.033
p-adj = 0.063
Ftl1
Fth1
Rps26
Rps2
Cox6a1
Cox5b
Tum_Ftl1
Rank in Gene List
Tum_Cpe

Figure S15. Identification of ambient RNA-high and low-quality proximal tubule and tumor cell clusters.
A) Left, UMAP of non-immune cells from 4 normal kidney samples prior to removal of ambient RNA-high clusters, PT_A and PT_B cell groupings labeled with dotted line. Right, heatmaps in UMAP space showing expression of pan-PT, S1 PT, S2 PT, and S3 PT markers. B) Violin plots showing number of features per cell, number of counts per cell, percentage of counts from *Rps* genes, percentage of counts from *Rpl* genes in PT_A and PT_B cells. C) GSEA results showing enrichment of top genes in low-RNA droplets shared across 4 normal kidney samples between PT_A and PT_B cells. D) UMAP of non-immune cells from 4 Cre-less AAV tumor samples prior to removal of the ambient RNA-high cluster. E) Violin plots showing number of features per cell, number of counts per cell, percentage of counts from *Rps* genes, percentage of counts from *Rpl* genes in tumor clusters. F) GSEA results showing enrichment of top genes in low-RNA droplets shared across 4 Cre-less AAV samples between Tumor_Ftl1 and Tumor_Neat1, Tumor_Ftl1 and Tumor_Cpe, and Tumor_Cpe and Tumor_Neat1.

Combo	Mouse Strain	Cre	Guide 1	Guide 2	Guide 3	Guide 4	Mice Injected	Kidney Tumors
1	B6 LSL-Cas9	Ksp-Cre (AAV)	VHL	TSC1	SETD2	PBRM1	25	0
2	B6,129 PBRM1 f/f, LSL-Cas9	Ksp-Cre (AAV)	VHL	TSC1	SETD2	CDKN2A	5	0
3	B6,129 PBRM1 f/f, LSL-Cas9	Ksp-Cre (AAV)	VHL	TSC1	SETD2	NT Control	9	0
4	B6,129 PBRM1 f/f, LSL-Cas9	Ksp-Cre (AAV)	VHL	TSC1	SETD2	HIF1a	10	0
5	B6,129 PBRM1 f/f, LSL-Cas9	Ksp-Cre (AAV)	VHL	TSC1	SETD2	EPAS1	7	0
6	B6 LSL-Cas9, Pax8-Cre	Pax8-Cre (Tg)	VHL	TSC1	SETD2	NT Control	7	0
7	B6 LSL-Cas9, Pax8-Cre	Pax8-Cre (Tg)	VHL	TSC1	SETD2	PBRM1	7	0
8	B6 LSL-Cas9, Pax8-Cre	Pax8-Cre (Tg)	VHL	TSC1	SETD2	BAP1	7	0
9	B6 LSL-Cas9, Pax8-Cre	Pax8-Cre (Tg)	VHL	TSC1	PBRM1	NT Control	25	0
10	B6 LSL-Cas9, Pax8-Cre	Pax8-Cre (Tg)	VHL	KEAP1	PBRM1	NT Control	25	0
11	B6 LSL-Cas9, Pax8-Cre	Pax8-Cre (Tg)	VHL	KEAP1	NT Control	NT Control	25	1
12	B6 LSL-Cas9, Pax8-Cre	Pax8-Cre (Tg)	VHL	KEAP1	TSC1	NT Control	25	24

Table S1. Guide RNA combinations tested for tumor development in the KSP-Cre and Cre-less AAV models

**Group**	**An #**	**Vhl (% edited)**	**Pbrm1 (% edited)**	**Keap1 (% edited)**	**Tsc1 (% edited)**
1-anti-PD1	689	38.83	21.49	40.59	29.51
1-anti-PD1	652	28.54	24.53	36.70	15.91
1-anti-PD1	1031	70.11	34.81	73.30	71.89
1-anti-PD1	1154	53.12	19.11	83.13	81.97
1-anti-PD1	1035	61.72	15.56	74.83	66.72
1-anti-PD1	975	73.73	33.90	74.92	71.43
1-anti-PD1	1061	76.61	31.99	79.13	57.01
2-anti-CTLA4	632	68.63	65.27	2.62	68.67
2-anti-CTLA4	750	17.46	47.08	85.82	85.85
2-anti-CTLA4	753	51.15	18.09	55.79	40.72
2-anti-CTLA4	989	44.94	7.72	58.43	58.93
2-anti-CTLA4	1109	78.25	73.39	77.60	80.27
2-anti-CTLA4	1012	73.51	11.00	83.72	84.61
2-anti-CTLA4	955	55.59	2.97	59.14	77.00
2-anti-CTLA4	1108_T1	76.60	0.37	78.57	77.92
3-PT2399	656	78.49	18.89	80.68	73.69
3-PT2399	677	74.22	10.63	81.69	83.16
3-PT2399	767	84.19	0.15	82.68	83.39
3-PT2399	746	57.86	15.00	44.48	56.19
3-PT2399	1032	57.04	15.22	70.36	55.88
3-PT2399	894	82.46	0.17	14.29	81.74
3-PT2399	995	69.55	37.74	78.57	75.93
3-PT2399	1079	50.73	47.96	74.13	54.90
3-PT2399	1013	61.07	10.53	67.50	62.14
3-PT2399	1015	71.84	57.06	75.41	73.00
3-PT2399	968	78.15	39.64	81.87	88.79
4-anti-PD1 &PT2399	673	4.17	5.97	78.61	80.10
4-anti-PD1 &PT2399	700	51.63	41.56	77.91	71.22
4-anti-PD1 &PT2399	1104	66.59	10.68	71.55	64.74
4-anti-PD1 &PT2399	1020	43.87	9.51	65.83	64.60
4-anti-PD1 &PT2399	1141	44.95	11.68	76.81	71.31
4-anti-PD1 &PT2399	1142_T1	49.46	1.55	43.47	54.51
4-anti-PD1 &PT2399	1083	54.41	52.04	68.85	64.28
4-anti-PD1 &PT2399	1100	59.66	39.89	53.53	60.34
4-anti-PD1 &PT2399	1085	70.62	24.44	62.26	72.69
4-anti-PD1 &PT2399	1089 T1	66.40	26.79	74.84	60.81
5-anti-CTLA4 & PT2399	613	63.23	20.72	54.02	64.23
5-anti-CTLA4 & PT2399	676	74.99	30.54	74.66	78.64
5-anti-CTLA4 & PT2399	749	48.15	0.75	58.69	43.78
5-anti-CTLA4 & PT2399	997T1	62.61	15.95	70.06	70.15
5-anti-CTLA4 & PT2399	655	67.20	0.52	94.20	40.81
5-anti-CTLA4 & PT2399	1016 T1	3.02	1.63	87.39	90.37
5-anti-CTLA4 & PT2399	1016 T2	68.02	59.05	69.38	57.29
5-anti-CTLA4 & PT2399	1036	56.19	33.53	73.26	72.01
5-anti-CTLA4 & PT2399	1152	79.54	3.24	75.77	83.61
5-anti-CTLA4 & PT2399	1076	75.22	57.17	80.15	75.15
5-anti-CTLA4 & PT2399	1054	75.11	0.19	82.49	84.12
5-anti-CTLA4 & PT2399	1081	57.72	42.07	56.34	49.62

6-anti-CTLA4&anti-PD1	755	51.03	13.45	49.60	49.44
6-anti-CTLA4&anti-PD1	626	70.03	72.86	71.72	69.48
6-anti-CTLA4&anti-PD1	692	75.92	3.13	81.34	68.79
6-anti-CTLA4&anti-PD1	952	91.97	0.15	0.00	63.37
6-anti-CTLA4&anti-PD1	956	56.23	16.68	66.18	58.75
6-anti-CTLA4&anti-PD1	962	55.09	22.87	71.25	69.33
6-anti-CTLA4&anti-PD1	990	62.91	14.99	64.50	59.46
6-anti-CTLA4&anti-PD1	1097	58.36	29.48	67.39	58.92
6-anti-CTLA4&anti-PD1	1053	69.62	1.55	72.12	75.71
7-anti-CTLA4, anti-PD1 & PT2399	630	60.56	52.31	65.14	64.36
7-anti-CTLA4, anti-PD1 & PT2399	629	27.37	0.74	46.85	49.03
7-anti-CTLA4, anti-PD1 & PT2399	670	76.88	21.37	81.43	82.91
7-anti-CTLA4, anti-PD1 & PT2399	790	45.96	15.69	58.28	48.91
7-anti-CTLA4, anti-PD1 & PT2399	747	81.54	0.13	79.51	79.91
7-anti-CTLA4, anti-PD1 & PT2399	960	1.08	1.29	90.50	83.19
7-anti-CTLA4, anti-PD1 & PT2399	1146 T1	48.96	18.42	49.54	38.99
7-anti-CTLA4, anti-PD1 & PT2399	1004	53.05	38.83	59.87	51.97
7-anti-CTLA4, anti-PD1 & PT2399	1150 T2	80.89	51.27	78.11	76.47
7-anti-CTLA4, anti-PD1 & PT2399	1017	43.90	30.70	47.45	48.97
7-anti-CTLA4, anti-PD1 & PT2399	1137T1	70.12	2.69	72.69	75.99
8-vehicles	581	42.42	16.64	47.49	45.16
8-vehicles	631	37.81	9.84	58.99	47.92
8-vehicles	658	47.18	28.09	56.20	45.95
8-vehicles	762	56.37	34.81	62.06	24.51
8-vehicles	768	62.77	51.37	66.95	52.05
8-vehicles	1093	75.10	8.28	81.76	82.06
8-vehicles	1021	56.99	2.13	55.44	59.06
8-vehicles	1030	49.77	15.80	53.20	0.37
8-vehicles	1074	37.75	23.88	44.30	39.44
8-vehicles	1071T1	50.71	30.12	61.08	45.56
8-vehicles	1102	27.34	17.35	27.94	21.05
8-vehicles	971	36.25	0.30	61.60	51.97

Table S2. Percent gene editing in tumors from multi-drug study (n=80)

Sample	Total yield (Mbases)	% >= Q30 bases	Total input reads	Total mapped reads	% Total mapped reads
Mus_T1	194,077	88.16	1,285,276,790	1,278,784,137	99.49
Mus_T2	188,367	90.21	1,247,458,464	1,242,576,739	99.61
Mus_T3	202,206	89.29	1,339,113,062	1,333,529,515	99.58
Mus_T4	183,063	89.93	1,212,341,102	1,207,673,088	99.61
Mus_T5	215,475	89.88	1,426,986,492	1,420,782,072	99.57
Mus_T6	199,441	90.57	1,320,801,174	1,315,173,240	99.57
Mus_T11	202,111	89.6	1,338,478,416	1,333,191,248	99.6
Mus_T8	182,611	90.31	1,209,343,804	1,205,204,920	99.66
Mus_T9	211,563	89.43	1,401,079,594	1,395,582,330	99.61
Mus_T10	194,817	90.37	1,290,180,780	1,285,655,919	99.65
Mus_N1	210,906	90.24	1,396,722,796	1,391,664,983	99.64
Mus_N2	202,505	89.99	1,341,092,528	1,334,751,077	99.53
Mus_N3	223,074	89.93	1,477,313,986	1,471,843,366	99.63
Mus_N4	223,346	89.98	1,479,113,764	1,473,098,316	99.59
Mus_N5	206,395	89.89	1,366,857,196	1,361,179,570	99.58
Mus_N6	175,579	89.31	1,162,779,702	1,158,070,024	99.59

Sample	Uniquely mapped reads	% Uniquely mapped reads	Total number of duplicate reads	% Total number of duplicate reads	Mean of mapped coverage	Mean of insert size (bases)
Mus_T1	1,092,887,565	85.03	185,896,572	14.46	58.18	471.73
Mus_T2	1,100,039,159	88.18	142,537,580	11.43	59.27	466.32
Mus_T3	1,140,724,879	85.19	192,804,636	14.4	61.48	495.98
Mus_T4	1,025,207,269	84.56	182,465,819	15.05	55.23	453.1
Mus_T5	1,239,361,269	86.85	181,420,803	12.71	66.76	472.96
Mus_T6	1,200,935,973	90.92	114,237,267	8.65	64.86	475.08
Mus_T11	1,126,525,271	84.16	206,665,977	15.44	59.98	445.31
Mus_T8	1,033,880,400	85.49	171,324,520	14.17	55.11	462
Mus_T9	1,197,897,406	85.5	197,684,924	14.11	64.01	462
Mus_T10	1,105,367,010	85.68	180,288,909	13.97	59.12	458
Mus_N1	1,179,458,560	84.44	212,206,423	15.19	62.88	471.85
Mus_N2	1,196,155,934	89.19	138,595,143	10.33	64.49	457.77
Mus_N3	1,274,644,223	86.28	197,199,143	13.35	68.7	466.11
Mus_N4	1,259,759,762	85.17	213,338,554	14.42	67.89	460.67
Mus_N5	1,157,008,559	84.65	204,171,011	14.94	62.33	452.41
Mus_N6	989,686,180	85.11	168,383,844	14.48	53.3	457.25

Table S3. Sequencing statistics for WGS data set

SI References

1. M. D. Young *et al.*, Single-cell transcriptomes from human kidneys reveal the cellular identity of renal tumors. *Science* **36**, 594-599 (2018).
2. M. Buse *et al.*, Lineage tracing reveals transient phenotypic adaptation of tubular cells during acute kidney injury. *iScience* **27**, 109255 (2024).
3. S. J. Fleming *et al.*, Unsupervised removal of systematic background noise from droplet-based single-cell experiments using CellBender. *Nat Methods* **20**, 1323-1335 (2023).
4. S. L. Wolock, R. Lopez, A. M. Klein, Scrublet: Computational Identification of Cell Doublets in Single-Cell Transcriptomic Data. *Cell Syst* **8**, 281-291 e289 (2019).
5. Y. Hao *et al.*, Integrated analysis of multimodal single-cell data. *Cell* **184**, 3573-3587 e3529 (2021).
6. I. Korsunsky *et al.*, Fast, sensitive and accurate integration of single-cell data with Harmony. *Nat Methods* **16**, 1289-1296 (2019).
7. A. Ransick *et al.*, Single-Cell Profiling Reveals Sex, Lineage, and Regional Diversity in the Mouse Kidney. *Dev Cell* **51**, 399-413 e397 (2019).
8. M. S. Balzer, T. Rohacs, K. Susztak, How Many Cell Types Are in the Kidney and What Do They Do? *Annu Rev Physiol* **84**, 507-531 (2022).
9. J. Park *et al.*, Single-cell transcriptomics of the mouse kidney reveals potential cellular targets of kidney disease. *Science* **360**, 758-763 (2018).
10. C. Novella-Rausell, M. Grudniewska, D. J. M. Peters, A. Mahfouz, A comprehensive mouse kidney atlas enables rare cell population characterization and robust marker discovery. *iScience* **26**, 106877 (2023).
